# Abscisic acid regulates root growth under osmotic stress conditions via an interacting hormonal network with cytokinin, ethylene and auxin

**DOI:** 10.1111/nph.13882

**Published:** 2016-02-18

**Authors:** James H. Rowe, Jennifer F. Topping, Junli Liu, Keith Lindsey

**Affiliations:** ^1^ The Integrative Cell Biology LaboratorySchool of Biological and Biomedical SciencesDurham UniversitySouth RoadDurhamDH1 3LEUK

**Keywords:** abscisic acid (ABA), *Arabidopsis thaliana*, auxin, ethylene, hormonal crosstalk, osmotic stress, PIN proteins, root development

## Abstract

- Understanding the mechanisms regulating root development under drought conditions is an important question for plant biology and world agriculture.
- We examine the effect of osmotic stress on abscisic acid (ABA), cytokinin and ethylene responses and how they mediate auxin transport, distribution and root growth through effects on PIN proteins. We integrate experimental data to construct hormonal crosstalk networks to formulate a systems view of root growth regulation by multiple hormones.
- Experimental analysis shows: that ABA‐dependent and ABA‐independent stress responses increase under osmotic stress, but cytokinin responses are only slightly reduced; inhibition of root growth under osmotic stress does not require ethylene signalling, but auxin can rescue root growth and meristem size; osmotic stress modulates auxin transporter levels and localization, reducing root auxin concentrations; PIN1 levels are reduced under stress in an ABA‐dependent manner, overriding ethylene effects; and the interplay among ABA, ethylene, cytokinin and auxin is tissue‐specific, as evidenced by differential responses of PIN1 and PIN2 to osmotic stress.
- Combining experimental analysis with network construction reveals that ABA regulates root growth under osmotic stress conditions via an interacting hormonal network with cytokinin, ethylene and auxin.

## Introduction

Increasing food security for a growing global population is a major challenge facing humanity. Modulation of root system architecture is a key feature of plant responses to drought, potentially leading to yield benefits (Comas *et al*., 2013; Uga *et al*., 2013). Understanding the mechanisms regulating root development under drought conditions is therefore an important question for plant biology and world agriculture.

Soils form a complex environment, and roots under drought stress face multiple challenges that can alter their development. As well as osmotic stress, plants may also encounter reduced nutrient uptake and mechanical impedance (Alam, 1999; Whalley *et al*., 2005). Less clear are the mechanisms by which these stresses mediate developmental changes.

Classic studies have shown that abscisic acid (ABA) biosynthesis and accumulated concentrations increase under drought stress (Zhang & Davies, 1987), and this response pathway is conserved among vascular and nonvascular land plants, including bryophytes (Takezawa *et al*., 2015). Low concentrations of applied ABA or amounts of osmotic stress can increase root growth, whilst high values can inhibit growth. Other hormones also play roles under drought – perturbation of cytokinin, auxin or ethylene pathways can affect survival or development under osmotic stress (Tran *et al*., 2007; Nishiyama *et al*., 2011; Cheng *et al*., 2013; Shi *et al*., 2014; Cui *et al*., 2015; Kumar & Verslues, 2015). How ABA and osmotic stress interact with other hormones remains poorly defined (van der Weele *et al*., 2000; Liu *et al*., 2014).

Extensive research has been carried out to understand the crosstalk between ethylene and ABA. Ethylene‐deficient and ‐insensitive mutants display increased ABA biosynthesis and responses, but exhibit reduced ABA‐mediated inhibition of root growth (Beaudoin *et al*., 2000; Ghassemian *et al*., 2000; Cheng *et al*., 2009). Phenotypic analysis of ethylene and ABA mutants has revealed little crosstalk between the signalling pathways directly (Cheng *et al*., 2009), but ethylene regulates root growth by altering auxin transport and biosynthesis, and several auxin transport mutants show reduced sensitivity to ABA in root length assays (Ruzicka *et al*., 2007; Swarup *et al*., 2007; Thole *et al*., 2014).

Drought and ABA reduce *trans*‐zeatin‐type cytokinin concentrations by modulating expression of cytokinin biosynthetic/metabolic enzymes (Dobra *et al*., 2010; Nishiyama *et al*., 2011). Moreover, it is known that cytokinin can inhibit auxin biosynthesis (Nordstrom *et al*., 2004) and promote ethylene biosynthesis (Vogel *et al*., 1998; Stepanova *et al*., 2007). Furthermore, ethylene promotes auxin biosynthesis (Ruzicka *et al*., 2007; Stepanova *et al*., 2007) and auxin can induce a rapid down‐regulation of cytokinin biosynthesis (Nordstrom *et al*., 2004; Jones & Ljung, 2011). It is also known that ethylene and cytokinin concentrations, and expression of the associated regulatory and target genes, are interlinked (To *et al*., 2004; Shi *et al*., 2012). Therefore, there will also be an interplay between the effects of osmotic stress on cytokinin biosynthesis and auxin and ethylene signalling. In addition, cytokinin‐deficient or ‐insensitive mutants display reduced ABA concentrations but increased ABA sensitivity, and drought induction of ABA biosynthesis has been shown to be similar to wild‐type (Nishiyama *et al*., 2011).

Therefore the metabolic and signalling responses of ABA, auxin, cytokinin and ethylene all play their roles in developmental changes effected by osmotic stress. Previously, we have constructed a network describing the interactions among auxin, ethylene, cytokinin and the POLARIS peptide (PLS) (required for correct auxin, ethylene and cytokinin signalling in Arabidopsis; Casson *et al*., 2002; Chilley *et al*., 2006), revealing a hormonal crosstalk circuit that regulates root growth (Liu *et al*., 2010). This model has been expanded to include auxin transport via the PIN‐FORMED (PIN) efflux transporters (Paponov *et al*., 2005) and has been implemented into a spatiotemporal model, which can reproduce the patterning of various hormones and response genes (Liu *et al*., 2013; Moore *et al*., 2015). In brief, our previous research has shown that Arabidopsis root development and response under standard laboratory growth conditions involves a complex hormonal crosstalk network of overlapping interactions among auxin, ethylene and cytokinin (Liu *et al*., 2010, 2013, 2014; Moore *et al*., 2015). One of the important properties of hormonal crosstalk in root development is that a change in one signalling component leads to changes in other signalling components.

Therefore, in order to understand the roles of plant hormones in root development, one of the key questions to address is how hormone concentrations and the expression of associated regulatory and target genes are mutually related. For example, we investigated how the crosstalk among auxin, ethylene and cytokinin is established via the function of the *PLS* gene. We showed that crosstalk between hormones occurs in that, in the *pls* mutant, auxin concentrations are reduced, cytokinin concentrations increased and ethylene remains approximately unchanged. Moreover, increasing the concentration of either ethylene or cytokinin inhibits *PLS* gene expression, while increasing auxin concentrations promotes *PLS* gene expression (Casson *et al*., 2002; Chilley *et al*., 2006). This example clearly demonstrates that auxin, ethylene, cytokinin, and *PLS* gene functions are interrelated. Although we have previously demonstrated how Arabidopsis root development is regulated by hormonal pathways exhibiting crosstalk (Liu *et al*., 2010, 2013, 2014; Moore *et al*., 2015), the crosstalk network we previously developed does not include the effects of osmotic stress.

Here, we examine the effect of osmotic stress on ABA, cytokinin and ethylene responses and how they mediate auxin transport, distribution and root growth through effects on PIN proteins. We show that under osmotic stress, Arabidopsis seedlings display increased ABA responses, and demonstrate the effects on auxin transport to the primary root meristem through altered PIN1 levels.

We then use this information to construct a new network to integrate the effects of osmotic stress and ABA with auxin, ethylene and cytokinin. This network develops novel insights into how an integrated system of ABA, auxin, ethylene and cytokinin is formed as a result of the repression of ethylene effects by ABA to limit auxin accumulation in the meristem. This brings new understanding to the control of root development under stress.

## Materials and Methods

### Plant material

*Arabidopsis thaliana* wild‐type seeds were from laboratory stocks of the Columbia (Col‐0) or C24 ecotypes, originally obtained from Lehle Seeds (Round Rock, TX, USA). *polaris (pls)* mutant seeds were previously generated by GUS promoter trapping in the C24 background (Topping *et al*., 1994; Topping & Lindsey, 1997). *proPLS::PLS:GFP* and *35S::PLS* seeds (*PLSox*) in Col‐0 background were previously generated by floral dipping (Casson *et al*., 2002).

*pDR5rev::3xVENUS‐N7* (Heisler *et al*., 2005), *35S::DII‐VENUS‐N7* (Brunoud *et al*., 2012) and *pTCS::GFP* (Muller & Sheen, 2008), all Col‐0 background, were obtained from the Nottingham Arabidopsis Stock Centre (NASC).

*proAUX1::AUX1‐YFP(116)* was obtained courtesy of Dr Ranjan Swarup (Nottingham University, UK).

*proPIN1::PIN1::GFP* (Benkova *et al*., 2003), *proPIN2::PIN2::GFP* (Xu & Scheres, 2005) and *proPIN4::PIN4::GFP* (Vieten *et al*., 2005) were obtained courtesy of Prof. Ben Scheres (Wageningen University, the Netherlands). *proARR5::GFP* and *proARR5::GUS* (Ws background) were obtained courtesy of Prof. Joseph Kieber (University of North Carolina, USA).

*proRGA::RGA::GFP* (Silverstone *et al*., 2001), Col‐0 background was obtained courtesy of Dr Ari Sadanandom (Durham University, UK).

### Plant growth conditions

Seeds were sterilized for 30 s with 70% (v/v) ethanol and 10 min with 20% commercial bleach containing 0.1% Tween‐20, then washed five times with sterile distilled water.

Seeds were placed on 10 cm round plates containing half‐strength Murashige and Skoog (MS) medium (2.2 g l^−1^; Sigma) with agar (5 g l^−1^; Sigma) and MES (6 mM, 1.2 g l^−1^; Sigma) and sealed with Micropore tape. To ensure simultaneous germination, seeds were stratified for 4–7 d at 4°C before transfer to a growth room (22°C, 18 h photoperiod). Plates were orientated horizontally except for root length assays, when they were orientated vertically.

Five days after germination (DAG), seedlings were transferred to poly(ethylene glycol) (PEG)‐infused half‐strength MS agar plates with water potentials (*ψ*_w_) of *c*. −0.14, −0.37 or −1.2 MPa, adapted from Verslues *et al*. (2006). The plates were sealed with Micropore tape and placed in a growth room.

### Preparation of PEG‐infused plates

The method is adapted from Verslues *et al*., (2006). Essentially an overlay solution containing PEG is poured over half‐strength MS agar plates and PEG is allowed to diffuse into the medium.

Both the agar medium and overlay solution contained half‐strength MS salts (2.2 g l^−1^) and MES buffer (6 mM, 1.2 g l^−1^) and were adjusted to pH 5.7 by adding 0.1 M KOH solution. High‐gel‐strength agar (5 g l^−1^; Melford Laboratories, Ipswich, UK) was added to the base medium before autoclaving. No sucrose was used, as it affects ABA signalling and to minimize the chance of bacterial/fungal contamination. After autoclaving, PEG‐8000 (Sigma) was added to the liquid overlay solutions depending on the desired osmotic pressure of the plate (0 g l^−1^ for −0.14 MPa, 250 g l^−1^ for −0.37 MPa, 550 g l^−1^ for −1.2 MPa). A quantity of 40 ml of medium was poured onto 10 cm square plates and allowed to set, after which 60 ml of the appropriate overlay solution was added. The plates were sealed with Parafilm, allowed to equilibrate for 15–24 h and the overlay solution removed before transferring seedlings and resealing with Micropore tape.

Medium water potentials were verified using a Wescor 5600 osmometer (ELITech, Berkhamsted, UK); the large sample chamber was used to allow direct measurements of solid medium. Osmolarity data were verified in 10 independent measurements for each treatment.

### RNA extraction and cDNA synthesis

Seedlings (100 mg; *c*. 30 seedlings at 5 or 6 DAG) were flash‐frozen in liquid nitrogen. The seedlings were ground on dry ice whilst still frozen and RNA was extracted using a Sigma Spectrum Total RNA kit (Sigma Aldrich), and DNase digestion was performed with the Sigma On‐column DNase kit (Sigma Aldrich). RNA concentration was determined with a Nanodrop ND1000 Spectrophotometer (ThermoFisher Scientific, Hemel Hempstead, UK).

Five nanograms of RNA in a 20 μl reaction mixture was used for cDNA synthesis, using the Invitrogen Superscript III First Strand Synthesis System (Invitrogen Ltd, Paisley, UK).

cDNA was diluted 1 : 4 for PCR and quantitative real‐time PCR (qPCR). cDNA was tested for genomic DNA contamination by PCR amplification of *ACT2*, using primers designed over an intron (Supporting Information Table S1). Samples contaminated with genomic DNA were treated with Promega RQ1 DNase, which was then denatured before the cDNA was synthesized again.

### Quantitative real‐time PCR (qPCR)

SYBR Green Jumpstart Taq Readymix (Sigma Aldrich) was used with a Corbett Scientific Rotorgene Q (Qiagen).

Expression of each gene was calculated using the Rotorgene Q Series software v.1.7, using the ΔΔCT method relative to expression of a paired reference gene amplification, according to the manufacturer's instructions. Amplification efficiencies of the genes of interest were checked to ensure they were all within 5% of the reference gene amplification efficiency. Melt curves were used to check for nonspecific/unwanted products and primer dimers. Stabilities of reference genes were verified by ΔΔCT comparison between all samples and the control. All sample amplifications were done in triplicate for technical repetition, with three biological replicates. AT5G15710 was selected as a reference gene, owing to its stable expression pattern under osmotic stress, under hormone applications and at various developmental stages (Czechowski *et al*., 2005). Primer sequences are listed in Table S1.

### Compound light microscopy

After 6 d on media containing combinations of PEG and indole‐3‐acetic acid (IAA), root tips were mounted in Hoyer's solution (Anderson, 1954) and imaged on a Zeiss Axioskop microscope (Carl Zeiss, Cambridge, UK), fitted with a Retiga 2000R camera (Photometrics, Marlow, UK) and using the ×20 Neoflu lens and differential interference contrast microscopy. At least three roots of each treatment were imaged, and the representative images were compiled in Gimp 2.8 (http://www.gimp.org).

### Confocal laser scanning microscopy

Before transferring to osmotic stress plates, plants were selected as being the same developmental stage and screened for fluorescence under a Leica stereo dissecting microscope with fluorescence (http://www.leica-microsystems.com). After 24 h osmotic treatment, roots were imaged. Whole seedlings were transferred to a propidium iodide solution (0.5 μg ml^−1^) for 1.5 min and washed for the same time in deionized water. Root tips were then removed with a razor blade and transferred to a slide. Roots were imaged with a Leica SP5 laser scanning confocal microscope. Gain, line averaging, detection frequencies and other microscope settings were altered between fluorescent marker lines to optimize image quality, but not between roots of the same marker line, to ensure comparability. Yellow fluorescent protein (YFP) was excited with the 514 nm band of the argon laser, green fluorescent protein (GFP) was excited with the 488 nm band of the argon laser and propidium iodide was excited at 548 nm. Sequential scans were used and detection spectra were optimized to minimize crossover between different fluorophores.

### Image analysis

Meristem size determinations and cell counts were performed using ImageJ (http://www.imagej.nih.gov/ij/). Meristem size was assayed by measuring the distance along the cell file from the quiescent centre to the first cell that is double the length of the previous cell.

Mean relative fluorescence was calculated with ImageJ for PIN1:GFP, proARR5::GFP, pTCS::GFP and DII:VENUS. CellSet (Pound *et al*., 2012) was used to measure PIN2:GFP and AUX1::YFP relative fluorescence. In quantifying fluorescence across replicate experiments (*n* = between 5 and 14, according to the experiment), individual dead cells were excluded to ensure data reflect hormonal outputs.

### Statistical analysis

All statistical tests were performed in Microsoft Excel 2010, using the Real Statistics add‐in (http://www.real-statistics.com/). The 0.05 level of significance was used.

## Results

### Osmotic stress inhibits primary root growth, modulated by ABA

**Figure 1:**
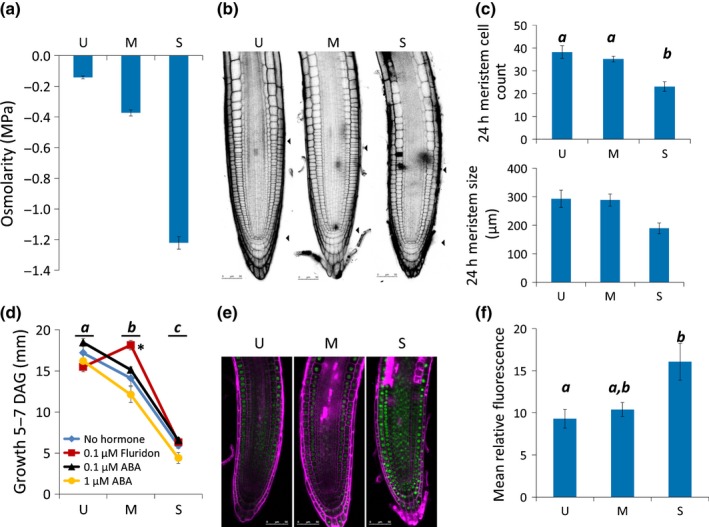
Experimental setup shows that osmotic stress leads to reduced Arabidopsis root growth, smaller meristem with fewer cells, and that abscisic acid (ABA) modulates root growth under stress, via increased DELLA. (a) Medium osmolarity of polyethylene glycol‐infused agar, measured with a vapour pressure osmometer 24 h after overlay solution is removed. *n *=* *10. (b) Primary root meristems stained with propidium iodide after 24 h osmotic stress treatment. Arrowheads indicate quiescent centre and approximate end of the meristematic zone. (c) Meristematic cell count (ANOVA *P *=* *0.002) and meristem size (ANOVA *P *=* *0.04) after 24 h osmotic stress treatment. (d) The effect of ABA and the ABA biosynthesis inhibitor fluridon on root growth under osmotic stress (treatment period, 5–7 d after germination (DAG)). Log_e_‐transformed two‐factor ANOVA:* P* (stress) < 0.0001, *P* (hormone) < 0.0001, *P* (interaction) = 0.0049. Blue diamonds, no hormone; black triangles, 0.1 μM ABA; yellow circles, 1 μM ABA; red squares, 0.1 μM fluridon. Asterisk indicates a significant effect. (e) proRGA::GFP:RGA under osmotic stress. (f) GFP:RGA fluorescence under osmotic stress. Measured in ImageJ, ANOVA,* P *=* *0.015. U, unstressed (−0.14 MPa); M, moderate stress (−0.37 MPa); S, severe stress (−1.2 MPa). For confocal images, scale bars represent 50 μm. Error bars indicate ± SEM. Lowercase letters indicate significance with a Tukey pairwise comparison.

Osmotic stress was induced by growing seedlings on half‐strength MS agar containing high‐molecular‐weight PEG (van der Weele *et al*., 2000; Verslues *et al*., 2006). This allowed us to examine the effects of osmotic stress independently of the ion stresses that mannitol/sorbitol/salt may cause or the mechanical impedance that can result from soil drying. Two stress treatments were chosen – a moderate stress (−0.37 MPa) and a severe stress (−1.2 MPa) – both of which were verified using a vapour pressure osmometer (Fig. 1a). Control plates lacking PEG were found to have an osmotic pressure of −0.14 MPa. Root cells are able to maintain a more negative water potential and cell turgor at moderate osmotic stress (−0.5 MPa) (Shabala & Lew, 2002) and root length assays demonstrated that plants were able to maintain at least some root growth under all three regimes.

As with previous studies, primary root growth was reduced under osmotic stress (Fig. 1d), and lateral root number was also adversely affected (Fig. S1; van der Weele *et al*., 2000; Deak & Malamy, 2005).

The effect of osmotic stress on primary root length is known to be modulated by ABA (Xiong *et al*., 2006). Low concentrations (0.1 μM) of exogenous ABA have a tendency to increase Arabidopsis primary root growth, whereas higher concentrations (> 1 μM) inhibit growth (Fig. 1d; Ghassemian *et al*., 2000). Inhibiting ABA biosynthesis with fluridon was found to rescue root elongation under moderate stress, suggesting that ABA is inhibiting root growth under stress.

Under osmotic stress, we observed a reduction in both meristem size and the number of cells in the primary root, which may be the cause of the reduction in growth (Fig. 1b,c). DELLA proteins such as RGA are inhibitors of growth and elongation, and are regulated by GA_3_, auxin, ethylene, ABA and stress, to modulate growth (Achard *et al*., 2003, 2006; Fu & Harberd, 2003). These proteins have been implicated in regulating meristem size and cell expansion in the elongation zone (Ubeda‐Tomás *et al*., 2008, 2009). To determine whether the effects of osmotic stress are mediated by signalling pathways rather than nonspecific cell damage effects, we used DELLA expression as a marker of growth‐related signalling changes in the root, by monitoring GFP:RGA expression in roots subjected to osmotic stress. As the root meristem becomes smaller with fewer cells as osmotic stress is increased, we found that GFP:RGA levels increased under stress (Fig. 1e,f). This evidence provides a link between osmotic stress and DELLA expression, and suggests that root growth is inhibited at the level of hormone signalling, rather than by a root elongation failure because of a lack of cell turgor or cell death.

### ABA‐dependent and ABA‐independent stress responses increase under osmotic stress, but cytokinin signalling responses have limited change

To verify that ABA‐dependent and ABA‐independent drought stress responses were active under our experimental osmotic stress regime, qPCR was carried out to monitor the expression of the genes *RD29B* and *DREB2B. RD29B* expression is highly ABA‐responsive but not responsive to ABA‐independent signalling, whereas *DREB2B* is inducible as an early response to dehydration but not to ABA treatment (Nakashima *et al*., 2000; Jia *et al*., 2012).

**Figure 2:**
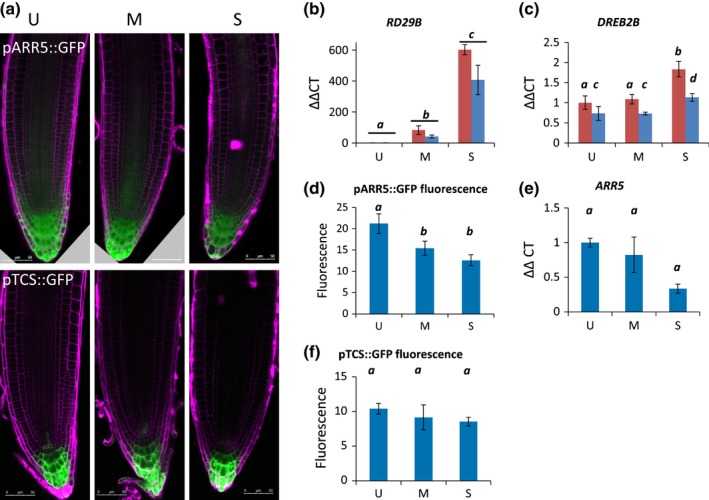
Arabidopsis abscisic acid (ABA)‐responsive genes (e.g. *RD29B*) and ABA‐independent stress genes (e.g. *DREB2B*) are up‐regulated by osmotic stress; cytokinin response genes (*ARR5*,*TCS*) may go down slightly. (a) proARR5::GFP (top panels) and pTCS::GFP (bottom panels) after 24 h osmotic stress treatment. (b) *RD29B* expression under osmotic stress. Log_e_‐transformed two‐factor ANOVA:* P* (stress) < 0.0001, *P* (time) = 0.42, *P* (interaction) = 0.15. Red, 6 h treatment; blue, 24 h treatment. (c) *DREB2B* expression under osmotic stress. Two‐factor ANOVA:* P* (stress) = 0.0014, *P* (time) = 0.0014, *P* (interaction) = 0.3. Red, 6 h treatment; blue, 24 h treatment. (d) *pARR5::GFP* fluorescence after 24 h osmotic stress treatment. Measured in ImageJ, ANOVA *P *=* *0.0015. (e) *ARR5* transcript abundance. (f) pTCS::GFP fluorescence after 24 h osmotic stress treatment, measured in ImageJ. ANOVA,* P *=* *0.44. U, unstressed (−0.14 MPa); M, moderate stress (−0.37 MPa); S, severe stress (−1.2 MPa). For confocal images, scale bars represent 50 μm. Error bars indicate ± SEM. Lowercase letters indicate significance with a Tukey pairwise comparison.

*RD29B* expression shows a very large (*c*. 100‐fold) increase under moderate and severe osmotic stress at 6 and 24 h (Fig. 2b). *DREB2B* expression increases significantly under severe stress at 6 h, but not under moderate stress, returning to near unstressed levels by 24 h (Fig. 2c). These results show that both osmotic treatments elicited expression changes in stress response genes.

The cytokinin receptor mutant *ahk3* maintains root growth under drought stress and altering cytokinin signalling/concentrations has been shown to alter survival of plants under drought (Tran *et al*., 2007; Werner *et al*., 2010; Kumar & Verslues, 2015). Therefore we also examined cytokinin responses under our specific osmotic stress treatments.

ARABIDOPSIS RESPONSE REGULATOR 5 (ARR5) is a type‐A negative regulator of cytokinin responses that displays increased expression under cytokinin treatment (Brandstatter & Kieber, 1998). Under osmotic stress treatment, we found that there was a small but statistically nonsignificant decrease in *ARR5* transcript abundance, although proARR5::GFP fluorescence decreased significantly (Fig. 2a,d,e). As *ARR5* expression can also be negatively regulated by ethylene, we also examined the responses of pTCS::GFP*,* a fluorescent protein under the control of a synthetic cytokinin responsive promoter (Muller & Sheen, 2008; Shi *et al*., 2012). *pTCS::GFP* expression showed a downward trend, but no statistically significant change, in fluorescence under stress (Fig. 2a,f). Therefore, under osmotic stress, Arabidopsis seedlings exhibit an increased ABA‐dependent and ‐independent stress responses, and possibly a small reduction in cytokinin responses.

### Inhibition of root growth under osmotic stress does not require ethylene signalling, but auxin can rescue root growth and meristem size

As both ethylene and auxin have been implicated in affecting survival and development under stress, we examined the growth responses of different ethylene and auxin mutants under osmotic stress. Auxin can either promote root growth by increasing meristem size or reduce root growth by inhibiting expansion in the elongation zone (Dello Ioio *et al*., 2008). Ethylene inhibits root growth by increasing auxin biosynthesis and basipetal auxin transport to the elongation zone via the efflux carrier PIN2 and influx carrier AUX1 (Ruzicka *et al*., 2007; Swarup *et al*., 2007).

**Figure 3:**
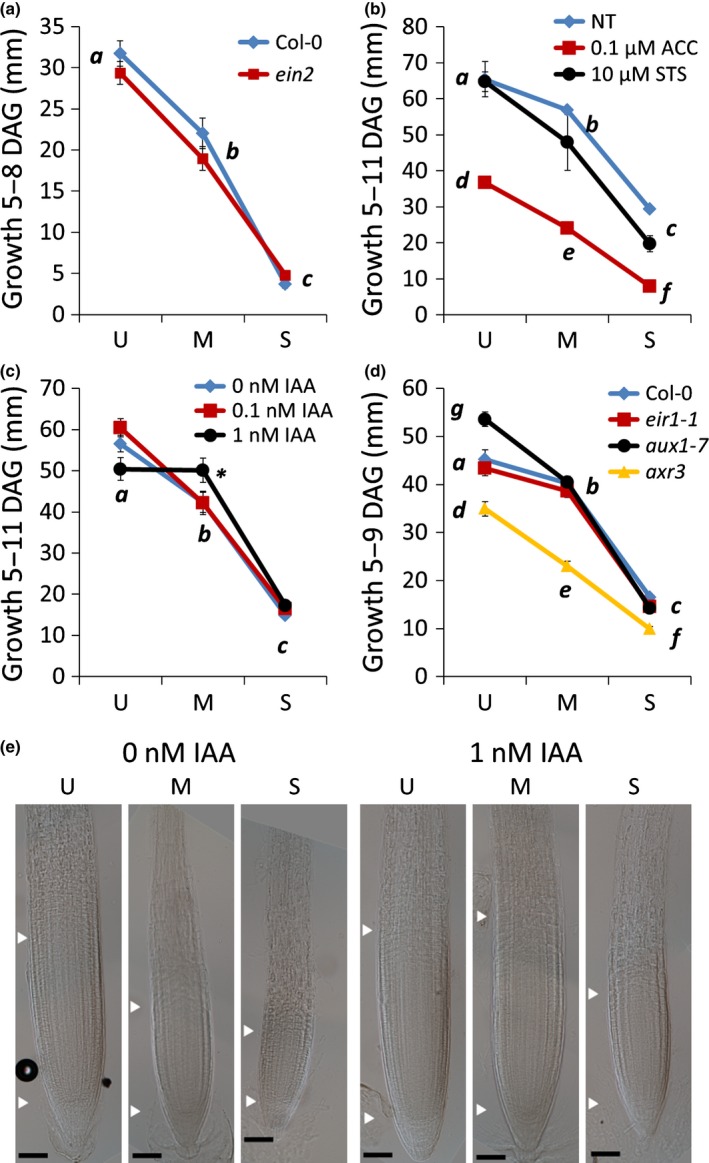
Auxin and ethylene regulation of Arabidopsis root length under osmotic stress. (a) Root growth of Col‐0 (blue diamonds) and the ethylene insensitive mutant *ein2* (red squares) under osmotic stress (treatment: 5–8 d after germination (DAG)) Log_e_‐transformed two‐factor ANOVA:* P* (stress) < 0.0001, *P* (mutant) = 0.71, *P* (interaction) = 0.063. (b) The effect of 1‐aminocyclopropane‐1‐carboxylic acid (ACC, red squares) and silver thiosulphate (STS, black circles) on root growth under osmotic stress, in Col‐0 (treatment: 5–11 DAG). Blue diamonds, no hormone treatment (NT). Two‐factor ANOVA:* P* (stress) < 0.0001, *P* (hormone) < 0.0001, *P* (interaction) = 0.12. (c) The effect of indole‐3‐acetic acid (IAA) on root growth under osmotic stress. Blue diamonds, no hormone treatment; red squares, 0.1 nM IAA; black circles, 1 nM IAA. Treatment: 5–11 DAG. Log_e_‐transformed two‐factor ANOVA:* P* (hormone) = 0.43, *P* (stress) < 0.0001, *P* (interaction) = 0.036. Asterisk indicates a significant effect. (d) The effect of osmotic stress on root growth on wild‐type (Col‐0, blue triangles), auxin transport mutants (*eir1‐1/pin2*, red squares; *aux1‐7*, black circles) and an auxin‐resistant mutant (*axr3‐1*, yellow triangles). Log_e_‐transformed two‐factor ANOVA:* P* (stress) < 0.0001, *P* (mutant) < 0.0001, *P* (interaction) < 0.0001. Treatment: 5–8 DAG. Error bars indicate ± SEM. (e) Root meristems under combined IAA and osmotic stress treatments (5–11 DAG). Arrowheads indicate the position of the quiescent centre and the end of the meristematic zone. U, unstressed (−0.14 MPa); M, moderate stress (−0.37 MPa); S, severe stress (−1.2 MPa). Bars, 50 μm. Lowercase letters (a‐d) indicate statistical significance.

EIN2 is required for ethylene responses (Guzman & Ecker, 1990) and AUX1 is required for auxin influx into cells (Swarup *et al*., 2001; Yang *et al*., 2006). *ein2*,* aux1‐7* and *eir1‐1/pin2* mutants display a similar reduction in primary root growth to wild‐type under osmotic stress (Fig. 3a,d). Supplementing growth medium with the ethylene precursor 1‐aminocyclopropane‐1‐carboxylic acid (ACC) was found to inhibit further wild‐type root growth, regardless of stress (Fig. 3b). This indicates that ethylene growth inhibition by basipetal auxin transport acts in a separate pathway to osmotic stress growth inhibition.

The reduction in root length, however, is not completely auxin‐independent. Supplementing growth medium with a low concentration of auxin (1 nM IAA) is mildly inhibitory to root growth (Evans *et al*., 1994). However, under moderate osmotic stress, this concentration of auxin was found to rescue root growth, and can partially rescue root growth under severe stress, suggesting that root length may be modulated through auxin responses under stress (Fig. 3c). This is supported by the observation that 1 nM IAA leads to a larger root meristem in roots subject to moderate and severe osmotic stress (Fig. 3e). These observations are also consistent with the growth responses of other auxin and ethylene mutants examined (Fig. S2). The *axr3‐1* line has reduced sensitivity to auxin (Leyser *et al*., 1996) and displays an exaggerated reduction in root growth and meristem size under osmotic stress (Figs 3d, S3). Other ethylene mutants such as *pls* and the *PLS* overexpressor line (PLSox) also display near wild‐type responses to osmotic stress (Fig. S2; Casson *et al*., 2002; Chilley *et al*., 2006).

**Figure 4:**
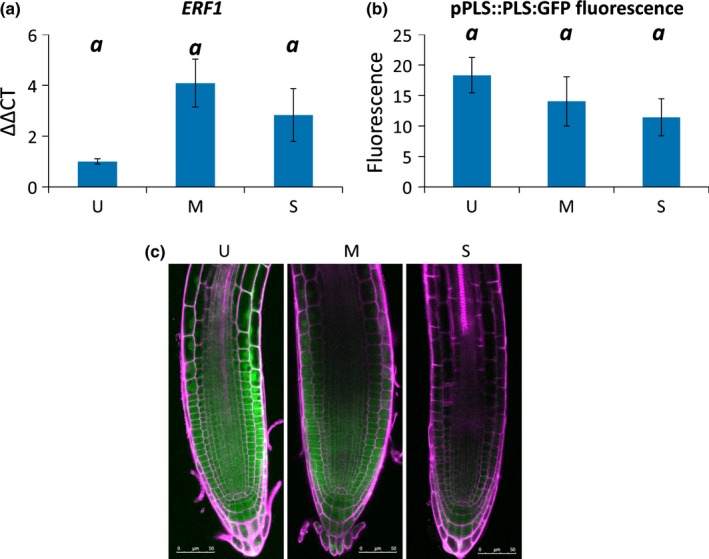
Ethylene response to osmotic stress in Arabidopsis. Osmotic stress causes an increased ethylene response, seen as increased expression of ethylene‐responsive genes (e.g *ERF1*) and suppression of genes down‐regulated by ethylene, such as *PLS*. (a) Relative transcript abundance of *ERF1* after 24 h osmotic stress treatment. ANOVA,* P *=* *0.09. (b) Relative fluorescence of proPLS::PLS:GFP after 24 h osmotic stress treatment. ANOVA,* P *=* *0.23. (c) proPLS::PLS:GFP after 24 h osmotic stress treatment. Green, green fluorescent protein; magenta, propidium iodide. Error bars ± SEM. Scale bars, 50 μm. U, unstressed (−0.14 MPa); M, moderate stress (−0.37 MPa); S, severe stress (−1.2 MPa). Letters indicate significance with a Tukey pairwise comparison.

Abiotic stresses, including osmotic stress and drought, can increase ethylene biosynthesis (Ichimura *et al*., 2000; Spollen *et al*., 2000; Joo *et al*., 2008), and various stress responses such as compatible solute accumulation and regulation of leaf growth are dependent on ethylene signalling (Skirycz *et al*., 2011; Cheng *et al*., 2013; Cui *et al*., 2015). To determine whether ethylene responses were altered under the osmotic stress conditions applied, we monitored expression of two genes associated with ethylene signalling, *ERF1* and *PLS*. *ERF1* expression is activated directly by ethylene signalling (Solano *et al*., 1998). Its expression increases under many abiotic stresses, and it can bind to GCC and dehydration‐responsive (DRE) promoter elements to activate stress responsive gene expression (Cheng *et al*., 2013). Our results show a trend of increased level of *ERF1* expression under moderate stress (ANOVA, *P *=* *0.09; Fig. 4a). *PLS* transcription has previously been shown to increase under auxin treatment and decrease in response to ACC treatment (Casson *et al*., 2002; Chilley *et al*., 2006). Under increasing osmotic stress, our results show a trend of a reduction in PLS:GFP fluorescence intensities (Fig. 4b,c), indicating lower auxin or higher ethylene signalling or both in stressed root tips.

Several papers have recently implicated a role for auxin in drought resistance and growth responses (Xu *et al*., 2013; Shi *et al*., 2014), but the precise role of auxin transport and distribution in these responses is unclear. Auxin increases meristem size, promoting growth, whilst cytokinin antagonizes auxin signalling, reducing meristem size and increasing cell differentiation (Dello Ioio *et al*., 2007, 2008; Moubayidin *et al*., 2010). ABA has recently been shown to act in coordination with ethylene and auxin to affect root growth, requiring basipetal auxin transporters PIN2 and AUX1 to inhibit root growth (Thole *et al*., 2014). ABA decreases levels of *PLETHORA* (*PLT*) gene expression and levels of PIN1, PIN2 and AUX1 in a reactive oxygen species‐dependent manner, and the ABA‐responsive transcription factor ABI4 has been shown to down‐regulate PIN1 expression (Shkolnik‐Inbar & Bar‐Zvi, 2010; Yang *et al*., 2014). Meristem size is reduced under osmotic stress, as a result of premature differentiation in an ABA‐dependent manner (Ji & Li, 2014; Ji *et al*., 2014). As our results showed no detectable increase in cytokinin signalling in response to osmotic stress (Fig. 2), we hypothesized that the reduction in meristem size was a result of altered auxin concentrations.

**Figure 5:**
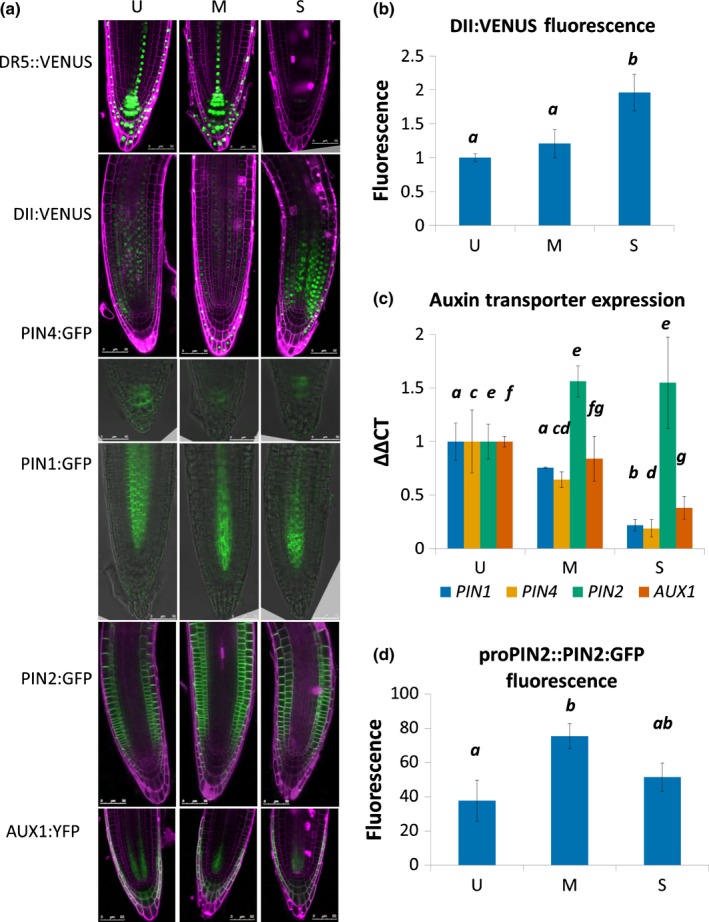
Response of auxin transport and responses to osmotic stress in Arabidopsis. Osmotic stress modulates auxin transporter levels, reducing root auxin concentrations. (a) pDR5rev::3xVENUS‐N7, 35S::DII:VENUS‐N7, proPIN4::PIN4:GFP, proPIN1::PIN1:GFP, proPIN2::PIN2:GFP and proAUX1::AUX1:YFP after 24 h osmotic stress treatment. (b) DII:VENUS fluorescence under osmotic stress. ANOVA,* P *=* *0.003. (c) Auxin transporter relative expression under osmotic stress. ANOVA: PIN1, *P *=* *0.05; PIN4, *P *=* *0.05; PIN2, *P *=* *0.33; AUX1, *P *=* *0.05. (d) proPIN2::PIN2:GFP fluorescence under osmotic stress. ANOVA,* P *=* *0.003. Lowercase letters indicate significance with a Tukey's pairwise comparison. Green, green fluorescent protein/yellow fluorescent protein; magenta, propidium iodide. Scale bars, 50 μm. Error bars indicate ± SEM. U, unstressed (−0.14 MPa); M, moderate stress (−0.37 MPa); S, severe stress (−1.2 MPa).

To examine the effect of osmotic stress on auxin distribution in the root, transgenic auxin biosensors and reporters were used. Under severe osmotic stress, the fluorescence of the *DR5::YFP* auxin sensor, which is activated in the presence of auxin (Sabatini *et al*., 1999; Heisler *et al*., 2005), decreased under osmotic stress (Fig. 5a), suggesting a reduced auxin response in the root tip. In agreement with this, *35S::DII:VENUS:N7*, which is rapidly degraded in the presence of auxin (Brunoud *et al*., 2012), was found to increase significantly in root tips under osmotic stress, indicating a decrease in root tip auxin signalling (Fig. 5a,b).

The auxin transporters PIN1 and PIN4 are localized to the membrane of the vascular tissues and root meristem, respectively, in *Arabidopsis* and funnel auxin from the stele into its concentration maxim around the quiescent centre and columella initials (Galweiler *et al*., 1998; Friml *et al*., 2002). We found that, following qPCR analysis, both *PIN1* and *PIN4* gene transcript abundances decreased under osmotic stress, with associated reductions in PIN1:GFP and PIN4:GFP fluorescence (Fig. 5a,c). PIN1::GFP also showed reduced polarity under osmotic stress and accumulated in bodies similar to brefeldin A bodies (Figs 5a, S4; Geldner *et al*., 2001). This is consistent with studies at the root apical meristem, where PIN1 internalization has been reported under a mannitol‐induced loss of turgor (Nakayama *et al*., 2012).

Under moderate osmotic stress, an increase in *PIN2* transcript and fluorescent protein abundances was observed, as found in previous studies (Fig. 5a,c,d; Xu *et al*., 2013). The auxin influx carrier AUX1, which is expressed in many of the same tissues as PIN2, showed decreased expression and fluorescence under osmotic stress (Fig. 5a,c).

These results suggest that a reduced auxin response in the root tip under osmotic stress, seen as reduced DR5::YFP and increased DII:VENUS expression, could be the consequence of altered PIN protein expression to limit auxin supply and remove auxin from the root meristem. Given that exogenous auxin application can rescue root growth under stress (Fig. 3), we investigated further the regulation of auxin accumulation and response in the root under osmotic stress.

### PIN1 levels are reduced under stress in an ABA‐dependent manner, overriding ethylene effects

Ethylene has been shown to increase, and ABA to repress, *PIN1* expression (Ruzicka *et al*., 2007; Shkolnik‐Inbar & Bar‐Zvi, 2010; Liu *et al*., 2013; Yang *et al*., 2014). As both ethylene and ABA biosynthesis increase under stress, we therefore decided to examine *PIN1* expression in the context of osmotic stress and these two hormones.

**Figure 6:**
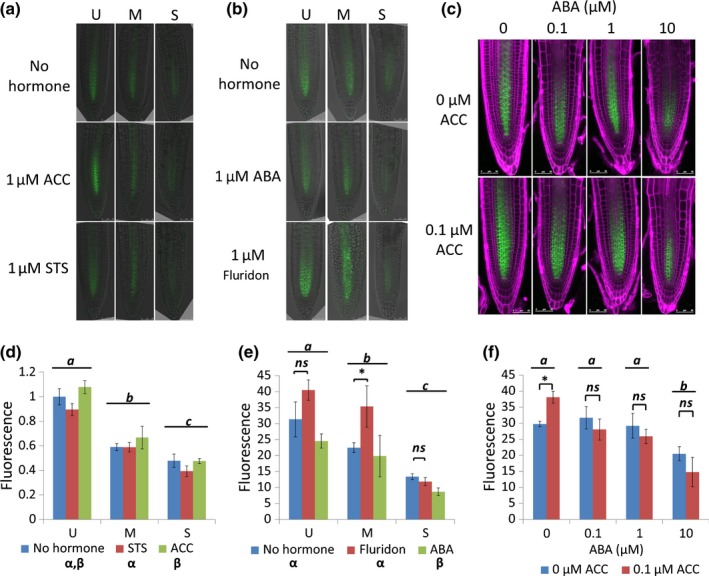
Relationship between osmotic stress, ABA and auxin/ethylene. ABA application reduces PIN1 expression further to osmotic stress, and overrides the effect of ethylene in increasing PIN1 levels, indicating that ABA suppresses the ethylene response in the Arabidopsis root. (a) proPIN1::PIN1:GFP under osmotic stress with either the ethylene precursor 1‐aminocyclopropane‐1‐carboxylic acid (ACC) or the perception inhibitor silver thiosulphate (STS). Green, green fluorescent protein (GFP). (b) proPIN1::PIN1:GFP under osmotic stress with either ABA or the biosynthesis inhibitor fluridon. Green, GFP. (c) proPIN1::PIN1:GFP under combined ACC and ABA treatment. Green is GFP fluorescence, magenta is propidium iodide fluorescence. (d) proPIN1::PIN1:GFP fluorescence under osmotic stress treatment with no hormone (blue bars), 1 μM ACC (green bars) or 10 μM STS (red bars). (e) proPIN1::PIN1:GFP fluorescence under osmotic stress treatment with either no hormone (blue bars), 1 μM ABA (green bars) or 1 μM fluridon (red bars). (f) proPIN1::PIN1:GFP fluorescence under combined ABA and ACC treatment. Error bars indicate ± SEM. U, unstressed (−0.14 MPa); M, moderate stress (−0.37 MPa); S, severe stress (−1.2 MPa). Lowercase letters indicate significance with a Tukey pairwise comparison; *ns*, no statistical difference; asterisk, a significant difference.

We found that pharmacological treatment of seedlings with the ethylene precursor ACC shows a trend of increasing PIN1:GFP levels, and application of the ethylene perception inhibitor silver thiosulphate (STS) leads to a trend of decreasing PIN1:GFP levels (*P* = 0.037), consistent with previous observations. However, as neither ACC nor STS treatment can rescue PIN1:GFP fluorescence under stress (Fig. 6a,d), the changes in PIN1:GFP levels under stress appear to be regulated independently of ethylene signalling in these conditions.

In proPIN1::PIN1:GFP transgenic seedlings, treatment with ABA led to decreased fluorescence, and treatment with the ABA biosynthesis inhibitor fluridon led to an increased PIN1 fluorescence (*P *<* *0.001; Fig. 6b,e), showing a down‐regulation of PIN1 fusion protein abundance by ABA. proPIN1::PIN1:GFP fluorescence was also affected by osmotic stress (*P *<* *0.0001), with increasing stress reducing PIN1 levels (Fig. 6b,e). Under moderate osmotic stress, fluridon treatment rescues PIN1 levels to untreated values (Fig. 6b,e), indicating a possible interaction between ABA signalling and osmotic stress to regulate PIN1 levels.

To determine whether ABA may therefore be overriding ethylene effects on PIN1 accumulation, the effects of combined hormone applications on PIN1::GFP fluorescence were determined. It was found that low concentrations (0.1–1 μM) of exogenous ABA had no significant effect on PIN1 levels, but higher concentrations (10 μM) reduced them significantly. Interestingly, it was also found that low exogenous concentrations of ABA were sufficient to suppress the high levels of PIN1:GFP fluorescence following ACC treatment to untreated values, indicating that ABA can override the effect of ACC on PIN1 levels (Fig. 6c,f).

In summary, this experimental analysis shows that: ABA‐dependent and ABA‐independent stress responses increase under osmotic stress, but cytokinin responses are only slightly reduced; inhibition of root growth under stress does not require ethylene signalling, but auxin can rescue root growth and meristem size; osmotic stress modulates auxin transporter levels and localization, reducing root auxin concentrations; PIN1 levels are reduced under stress in an ABA‐dependent manner, overriding ethylene effects; and the interplay of the four hormones (ABA, auxin, cytokinin and ethylene) is tissue‐specific. In particular, PIN1, which is expressed in the stele cells, and PIN2, which is expressed in the epidermis/cortex cells of the root, respond differentially to osmotic stress. Therefore, our experimental data indicate that an analysis of the regulation of root growth under osmotic stress requires a study of the interplay between ABA, auxin, ethylene and cytokinin as an integrative system.

### Constructing hormonal crosstalk networks to formulate a systems view of the regulation of root growth by multiple hormones under osmotic stress conditions

**Figure 7:**
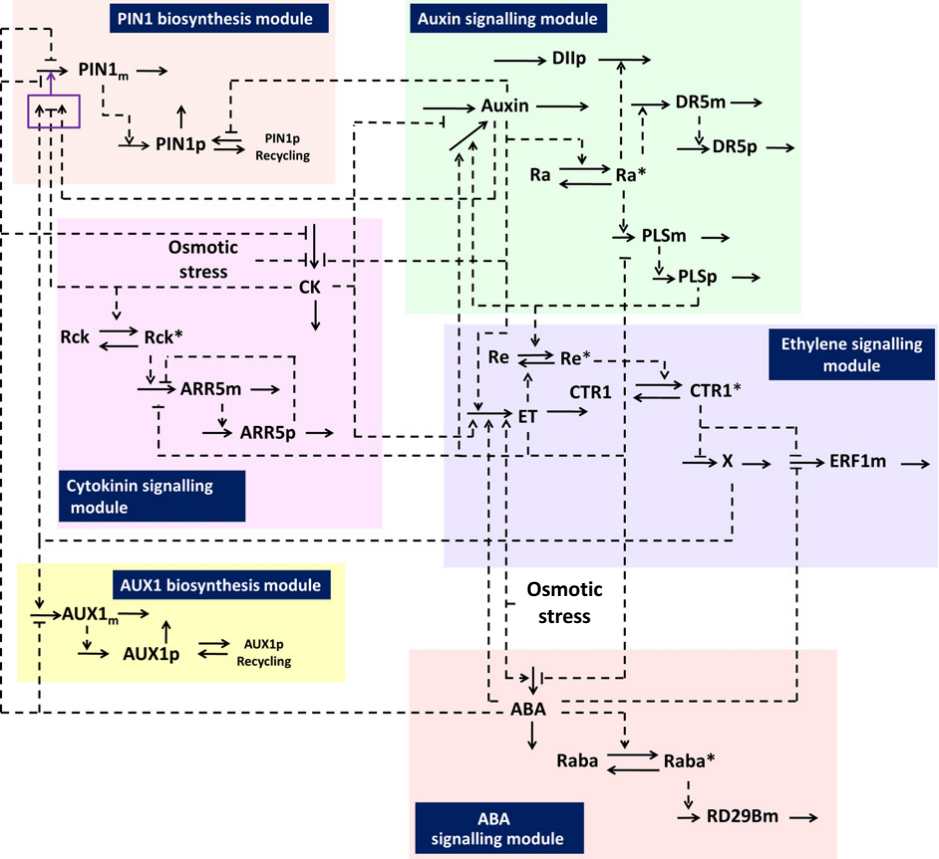
A hormonal crosstalk network for the regulation of root growth under osmotic stress conditions, in a vascular cell expressing PIN1, revealing that abscisic acid (ABA) regulates Arabidopsis root growth under osmotic stress conditions via an interacting hormonal network with cytokinin, ethylene and auxin. Abbreviations: Ra, inactive auxin receptor; Ra*, active auxin receptor; DR5m, *DR5* regulated *YFP* mRNA transcript; DR5p, *DR5* regulated yellow fluorescent protein; DIIp, DII‐VENUS protein; PIN1m, *PIN1* mRNA transcript; PIN1p, PIN1 auxin efflux transporter protein; AUX1m, AUX1 mRNA transcript; AUX1p, AUX1 auxin influx transporter protein; PLSm, *POLARIS* mRNA transcript; PLSp, POLARIS peptide; ET, ethylene; Re, inactive ethylene receptor; Re*, active ethylene receptor; CTR1, inactive CTR1 kinase; CTR1*, active CTR1 kinase; X, the unknown factor that regulates auxin transport from the aerial tissues; ERF1m, *ERF1* mRNA transcript ; Raba, inactive ABA receptor; Raba*, active ABA receptor; RD29Bm, *RD29B* mRNA transcript CK, active cytokinin; Rck, inactive cytokinin receptor; Rck*, active cytokinin receptor. ARR5m, *ARR5* mRNA transcript; ARR5p, ARR5 protein; osmotic stress, the osmotic stress imposed by the growth medium.

To understand better the relationships between the signalling pathways studied under osmotic stress, we developed a network approach, based on our experimental data and evidence in the literature (Fig. 7). The rationale for the network construction is described in Notes S1. Integration of available data reveals that ABA regulates root growth under osmotic stress conditions via an interacting hormonal network with cytokinin, ethylene and auxin. Although each hormone has its own signalling module to regulate its downstream gene expression, the signalling of four hormones (ABA, cytokinin, ethylene and auxin) exhibit interplay under osmotic stress conditions. The PIN auxin efflux carriers and influx carrier AUX1 also respond to osmotic stress, and therefore they play important roles in the interaction network. In addition, the interplay of the four hormones is tissue‐specific. In particular, PIN1, which is localized in the stele cells, and PIN2, which is localized in the epidermis/cortex cells, respond differentially to osmotic stress (Fig. 5). Therefore, regulation of root growth under osmotic stress conditions must be elucidated as an integrative hormonal crosstalk system in a tissue‐specific context.

We previously developed a hormonal interaction network for a single Arabidopsis cell by iteratively combining modelling with experimental analysis (Liu *et al*., 2010). We described how such a network regulates auxin concentration in the Arabidopsis root, by controlling the relative contribution of auxin influx, biosynthesis and efflux, and by integrating auxin, ethylene and cytokinin signalling. Recently, we have developed this hormonal interaction network to include PIN1 or PIN2 activities in a single Arabidopsis cell (Liu *et al*., 2013, 2014), and subsequently moved on to study the spatiotemporal dynamics of hormonal crosstalk in a multicellular root structure (Moore *et al*., 2015). Here we show that, after now incorporating ABA into the existing hormonal crosstalk network, a novel network for osmotic stress conditions can be constructed. Fig. 7 describes the interplay among ABA, cytokinin, ethylene, auxin, PIN1 and AUX1 in a single stele cell under osmotic stress conditions. Similarly, Fig. S5 describes the interplay among ABA, cytokinin, ethylene, auxin, PIN2 and AUX1 in a single epidermis/cortex cell under osmotic stress conditions.

The network reveals that under osmotic stress, owing to the promotion of biosynthesis or signalling of both ABA and ethylene, expression of *RD29B* and *ERF1* increases, as *RD29B* and *ERF1* expression is activated directly by ABA and ethylene signalling, respectively (Solano *et al*., 1998; Jia *et al*., 2012). Increasing ethylene biosynthesis promotes auxin biosynthesis (Swarup *et al*., 2001, 2007), which inhibits cytokinin biosynthesis (Nordstrom *et al*., 2004). However, because of the overriding role of ABA over the regulation of PIN1 by ethylene (Fig. 5), the regulation of PIN1 by auxin, ethylene and cytokinin is overridden by ABA under osmotic stress. Consequently, expression of PIN1 is lower under osmotic stress (Fig. 5).

However, this overriding ABA effect is tissue‐specific. In the epidermis/cortex, PIN2 expression increases under osmotic stress (Figs 5, S5). The decreased PIN1 and increased PIN2 expression reduce auxin concentrations in the root tip under osmotic stress conditions, and thus DII:VENUS levels increase despite the potential for ethylene to increase auxin accumulation. As auxin promotes and ethylene inhibits the expression of *PLS*, expression of *PLS* is lower under osmotic stress. As PLS in turn promotes auxin accumulation in the root tip (Liu *et al*., 2010, 2013), a decreased *PLS* expression correspondingly reduces auxin concentrations, and this effect is in addition to the effects of a decreased PIN1 expression and an increased PIN2 expression under osmotic stress. A decreased *PLS* expression also enhances the ethylene pathway (Casson *et al*., 2002; Chilley *et al*., 2006), promoting *ERF1* expression.

In addition, osmotic stress may inhibit cytokinin biosynthesis (Dobra *et al*., 2010; Nishiyama *et al*., 2011). It is known that cytokinin can inhibit auxin biosynthesis (Nordstrom *et al*., 2004) and promote ethylene biosynthesis (Vogel *et al*., 1998; Stepanova *et al*., 2007). Therefore, there is also an interplay between the effects of osmotic stress on cytokinin biosynthesis and auxin and ethylene signalling. Although exogenous application of ACC increases *AUX1* expression (Ruzicka *et al*., 2007), *AUX1* showed decreased expression and protein fusion fluorescence under osmotic stress. This implies that the increased ABA biosynthesis under osmotic stress plays an important role in *AUX1* expression and fluorescence. There is also an interplay between this reduced *AUX1* expression and fluorescence, and all components in the network (Fig. 7) as a result of the effects on the auxin concentrations in the root tip.

This work shows that combining experimental analysis with network construction reveals that ABA regulates root growth under osmotic stress conditions via an interacting hormonal network with cytokinin, ethylene and auxin. One of the important properties of this hormonal crosstalk network under osmotic stress conditions is that a change in one signalling component leads to changes in other signalling components. Therefore, elucidating the regulation of root growth under osmotic stress conditions requires the study of multiple hormones as an integrated system.

## Discussion

**Figure 8:**
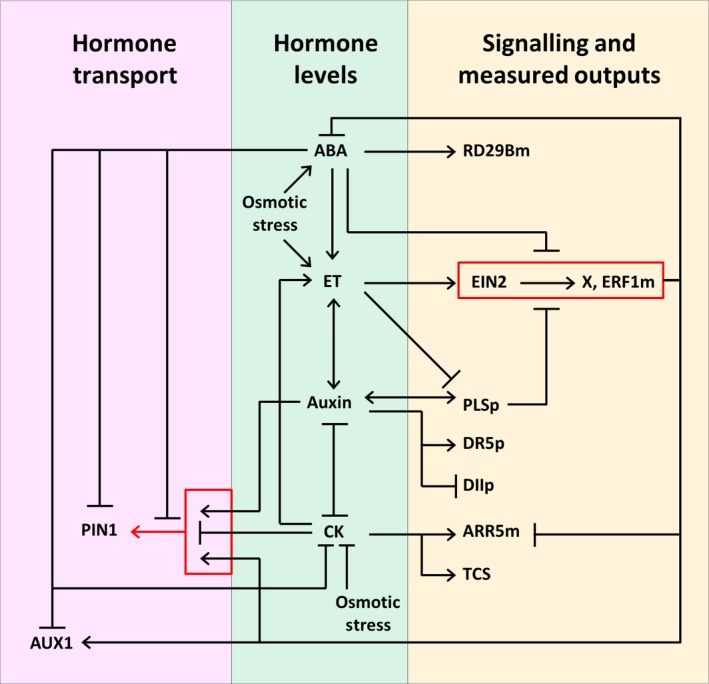
A simplified representation of the hormonal crosstalk network for the regulation of root growth under osmotic stress conditions, in a vascular cell expressing PIN1, demonstrating that the responses of auxin transporters, hormones and signalling components to osmotic stress are nonlinear and complex. Abbreviations: DR5p, *DR5* regulated yellow fluorescent protein; DIIp, DII‐VENUS protein; PLSp, POLARIS peptide; PIN1, PIN1 auxin efflux transporter protein; AUX1, AUX1 auxin influx transporter protein; ET, ethylene; X, the unknown factor that regulates auxin transport from the aerial tissues; EIN2, EIN2 ethylene signalling protein; ERF1m, *ERF1* mRNA transcript; ABA, abscisic acid; RD29Bm, *RD29B* mRNA transcript; CK, active cytokinin; ARR5m, *ARR5* mRNA transcript; TCS, cytokinin response reporter; osmotic stress, the osmotic stress imposed by the growth medium. Red boxes group related activities.

The hormonal crosstalk networks that we have developed (Figs 7, 8, S5, S6) describe the actions of multiple hormones and the associated regulatory and target genes under osmotic stress conditions. They provide a means to integrate our experimental analysis with a variety of experimental data in the literature. Such networks formulate a systems view on the regulation of root growth by multiple hormones under osmotic stress conditions. Specifically, the causal regulatory relationships of auxin efflux and influx transporters, concentrations of four hormones (ABA, auxin, ethylene and cytokinin), signalling components we have experimentally measured, and osmotic stress can be understood as an integrative system, as summarized in Figs 8 and S6. Figs 8 and S6 are the simplified descriptions of Figs 7 and S5, respectively. All these figures reveal the nonlinear and complex responses of auxin transporters, hormones and signalling components to osmotic stress.

Although ethylene‐induced basipetal auxin transport in the root is required for ABA to limit root growth under unstressed conditions (Beaudoin *et al*., 2000; Ghassemian *et al*., 2000; Thole *et al*., 2014), we have shown that osmotic stress limits root growth independently of this mechanism. We present the hypothesis that auxin transport to the root via PIN1 is limited under osmotic stress in an ABA‐regulated manner, and, together with enhanced PIN2 levels, leads to reduced auxin concentrations in the root meristem. Lower auxin concentrations lead to a reduction in meristem size and reduced root growth.

Cytokinin‐deficient plants display increased ABA sensitivity, but cytokinin receptor mutants show increased root growth under stress (Nishiyama *et al*., 2011; Kumar & Verslues, 2015). This would place cytokinin signalling downstream of ABA in regulating root growth under stress.

In Arabidopsis, the auxin : cytokinin ratio is critical in determining the rate of root growth. Cytokinin inhibits root growth by antagonizing auxin, to modulate the rate of cell division and differentiation in the root apical meristem (Dello Ioio *et al*., 2007, 2008; Moubayidin *et al*., 2010). As active cytokinin concentrations and cytokinin signalling are reduced under drought and osmotic stress (as indicated by the reduced expression of the cytokinin‐sensitive proARR5::GFP reporter; Fig. 2(c–e); Dobra *et al*., 2010; Nishiyama *et al*., 2011), but the meristem is smaller, it seems likely that meristem size is primarily regulated by altered auxin responses in these conditions. In cytokinin receptor mutants, auxin sensitivity would be predicted to increase, making plants more resistant to root growth inhibition as a result of reduced auxin concentrations. The combination of increased ABA sensitivity and enhanced root growth may account for the increase in drought stress tolerance of cytokinin‐deficient plants (Tran *et al*., 2007; Werner *et al*., 2010; Nishiyama *et al*., 2011).

Auxin application cannot completely rescue root growth under severe stress so factors other than auxin‐mediated regulation of meristem size may also be limiting growth. It is possible that at higher stress levels, cells exhibit reduced expansion as a result of reduced water availability, or that the high rate of programmed cell death is limiting growth (Duan *et al*., 2010). Under stress, plants must also divert significant resources to protective measures such as compatible solute accumulation, *LATE‐EMBRYOGENESIS‐ABUNDANT* (*LEA*) gene transcription and chaperone transcription, so constitutively drought‐tolerant plants often display dwarf phenotypes (Bray, 1997; Kasuga *et al*., 1999). It is possible that the balance of growth against protection may be playing a role here, limiting root growth indirectly.

Construction of hormonal crosstalk networks (Figs 7, 8, S5, S6) reveals multiple layers of complexity in the regulation of root development by osmotic stress. One layer of complexity is how hormone concentrations and the expression of their associated regulatory and target genes are mutually related. Another layer of complexity is how the interrelated hormones and gene expression quantitatively control root growth. Figs 7, 8, S5 and S6 show that the responses of auxin transporters, hormones and signalling components are linked via hormonal crosstalk networks. Therefore, a change in one response may lead to changes in other responses, and understanding the effects of one component (in Figs 7, 8, S5, S6) requires consideration of how this component affects all other components.

Experimentally, it has been shown that mutants in one PIN protein family member change the level or localization of other remaining PIN proteins under nonstressed growth conditions (Blilou *et al*., 2005). It has also been shown that *pin1* and *pin2* single mutants only display a moderate reduction of root length and root meristem size (Blilou *et al*., 2005). Our data here show that, under osmotic stress, PIN1 expression decreases and PIN2 expression increases. The decreased PIN1 and increased PIN2 expression work together to reduce auxin concentrations in the root tip. This example shows that change in auxin concentration cannot be attributed to the function of an individual PIN protein under osmotic stress. Although data in Figs 7, 8, S5 and S6 show how PIN1 and PIN2 link with ABA, auxin, ethylene and cytokinin under osmotic stress, the hormonal crosstalk for other PIN proteins currently cannot be established. This is because there is insufficient biological knowledge to establish the hormonal crosstalk for other PINs even if no osmotic stress exists.

Furthermore, in order to quantitatively link a mutant gene with root length or root meristem size control, any changes in the concentration of all relevant hormones must be quantitatively analysed. This is because ABA, auxin, ethylene and cytokinin are involved in root development and a mutant may change all or some of the four hormones to some extent. For example, establishment of a quantitative relationship between the *pin2* mutant and root length needs to establish not only a mathematical model for studying how the *pin2* mutant quantitatively affects other transporters and all four hormones via hormonal crosstalk networks (Figs 7, 8, S5, S6), but also the quantitative relationship between all hormones and root length by combining both experimental and modelling analysis. However, this is beyond the context of the current work.

The network we have constructed provides new insight into the interactions of phytohormones and how they regulate growth under stress. Based on experimental results (Nordstrom *et al*., 2004), our hormonal crosstalk networks (Figs 7, 8, S5, S6; Liu *et al*., 2013) describe a negative regulation of auxin biosynthesis by cytokinin. However, Jones *et al*. (2010) have shown that cytokinin positively regulates auxin biosynthesis in young developing tissues (10 DAG). In previous work, our hormonal crosstalk network analysis revealed that both sets of experimental results (Nordstrom *et al*., 2004; Jones *et al*., 2010) can be incorporated into the hormonal crosstalk network, leading to the same conclusions about other regulatory relationships of hormonal crosstalk (Liu *et al*., 2013). Hormonal crosstalk networks can also be constructed for the case where a positive regulation of auxin biosynthesis by cytokinin is described with all other regulatory relationships remaining unchanged.

As we have demonstrated, the network can be used to investigate how an integrated system of ABA, auxin, ethylene and cytokinin is formed under osmotic stress, as a result of the repression of ethylene effects by ABA via the enhanced transport of auxin away from the meristem and towards the elongation zone. Recently, we have shown that spatiotemporal modelling of hormonal crosstalk can simulate and explain the concentration and patterning of hormones and gene expression level in Arabidopsis wild‐type and mutant roots (Moore *et al*., 2015). However, that hormonal crosstalk does not include the effects of osmotic stress. Therefore, the novel hormonal crosstalk network developed in the current work provides a framework for spatiotemporal modelling of hormonal crosstalk under osmotic stress conditions, and will allow us to analyse how the patterning of multiple hormones regulates root development under osmotic stress. In particular, this will allow us to examine the mechanisms by which ABA could override ethylene induction of *PIN1* gene expression, whilst still allowing *PIN2* expression to increase.

The hormonal crosstalk network developed in this work will also allow us to further interrogate interactions with other growth‐regulating hormones such as the gibberellin (GA)/DELLA system. DELLA proteins are degraded as part of the GA signalling pathway and are viewed as master regulators of plant growth (Dill *et al*., 2001). Levels of the DELLA protein RGA increase under osmotic stress (Fig. 1) and ABA has previously been shown to increase RGA stability (Achard *et al*., 2006). High DELLA levels can reduce cell proliferation and the rate of differentiation to regulate meristem size (Ubeda‐Tomás *et al*., 2008, 2009; Achard *et al*., 2009). Several models already exist detailing how the GA signalling cascade is regulated by negative feedback loops and how hormone dilution can explain the cessation of cell expansion in the elongation zone (Band *et al*., 2012; Middleton *et al*., 2012). By further integrating other hormones into the network, we should in future be able to elucidate how ABA, cytokinin, ethylene, auxin and other hormones such as GA regulate root growth under osmotic stress.

## Author contributions

J.H.R., J.F.T., J.L. and K.L. devised the experiments and analysed the data; J.H.R. carried out the experimental work.

## Supporting information

Please note: Wiley Blackwell are not responsible for the content or functionality of any supporting information supplied by the authors. Any queries (other than missing material) should be directed to the *New Phytologist* Central Office.

**Fig. S1** Lateral root number is reduced under osmotic stress.
**Fig. S2** The ethylene oversensitive mutant *polaris* (*pls*) displays similar root growth responses to wild‐type (C24) under osmotic stress treatment.
***Fig. S3** Arabidopsis mutant meristems after 4 d of osmotic stress treatment.*
***Fig. S4** PIN1 localization changes under osmotic stress.*
***Fig. S5** A hormonal crosstalk network for the regulation of root growth under osmotic stress conditions, in an epidermis/cortex cell expressing PIN2.*
***Fig. S6** A simplified schematic for the hormonal crosstalk network for the regulation of root growth in an epidermis/cortex cell expressing PIN2.*
***Table S1** Primer sequences*
**Notes S1** Construction of hormonal crosstalk networks under osmotic stress conditions.
