# Supplementary material for: Abscisic acid regulates root growth under osmotic stress conditions via an interacting hormonal network with cytokinin, ethylene and auxin
Source: New Phytol. 2016 Feb 18;211(1):225–39. doi: 10.1111/nph.13882 (PMC4982081; doi:10.1111/nph.13882)
Supplement: Supplementary file 1 — Fig. S1 Lateral root number is reduced under osmotic stress. Fig. S2 The ethylene oversensitive mutant polaris (pls) displays similar root growth responses to wild‐type (C24) under osmotic stress treatment. Fig. S3 Arabidopsis mutant meristems after 4 d of osmotic stress treatment. Fig. S4 PIN1 localization changes under osmotic stress. Fig. S5 A hormonal crosstalk network for the regulation of root growth under osmotic stress conditions, in an epidermis/cortex cell expressing PIN2. Fig. S6 A simplified schematic for the hormonal crosstalk network for the regulation of root growth in an epidermis/cortex cell expressing PIN2. Table S1 Primer sequences Notes S1 Construction of hormonal crosstalk networks under osmotic stress conditions. [file NPH-211-225-s001.pdf]

New Phytologist

***New Phytologist* Supporting Information**

Article title: **Abscisic acid regulates root growth under osmotic stress conditions via an interacting hormonal network with cytokinin, ethylene and auxin**

Authors: James H. Rowe, Jennifer F. Topping, Junli Liu and Keith Lindsey

Article acceptance date: 6 January 2016

The following Supporting Information is available for this article:

Fig. S1 Lateral root number is reduced under osmotic stress.

Fig. S2 The ethylene oversensitive mutant *polaris (pls)* displays similar root growth responses to wildtype (C24) under osmotic stress treatment.

Fig. S3 Arabidopsis mutant meristems after 4 d of osmotic stress treatment.

Fig. S4 PIN1 localisation changes under osmotic stress.

Fig. S5 A hormonal crosstalk network for the regulation of root growth under osmotic stress conditions, in an epidermis/cortex cell expressing PIN2.

Fig. S6 A simplified schematic for the hormonal crosstalk network for the regulation of root growth in an epidermis/cortex cell expressing PIN2.

Table S1 Primer sequences.

Notes S1 Construction of hormonal crosstalk networks under osmotic stress conditions.

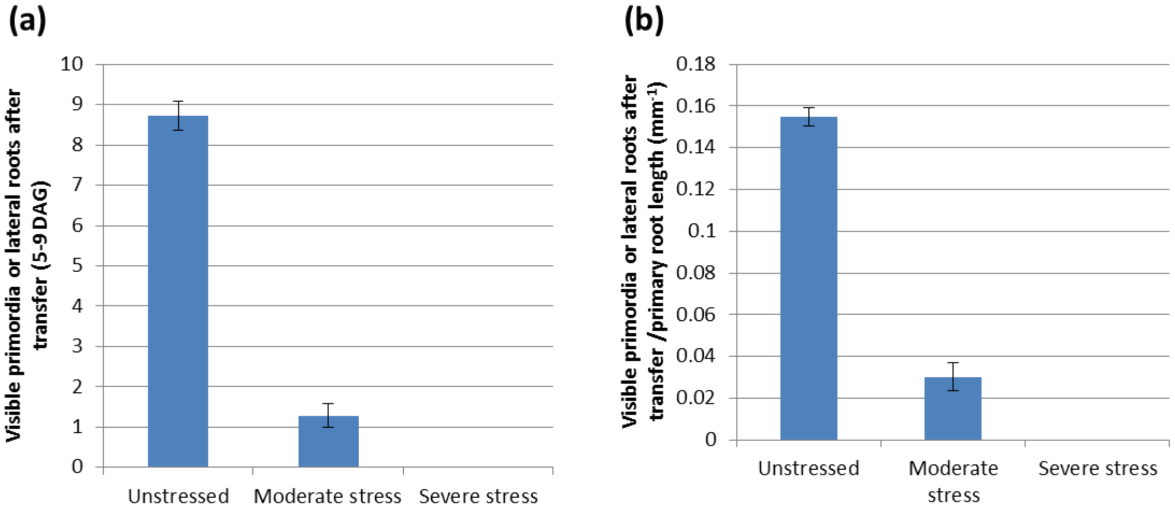

Fig. S1 Lateral root number is reduced under osmotic stress

(a) Total visible lateral roots and primordia formed after transfer to osmotic stress media.

(b) Visible lateral roots and primordia/primary root length.

Col-0 seedlings were transferred from 1/2MS plates to 1/2MS + Poly(ethylene glycol) mol wt. 8000 at 5 d after germination (DAG). After 4 d treatment, plates were digitised with a flatbed scanner and imageJ was used to count lateral roots.

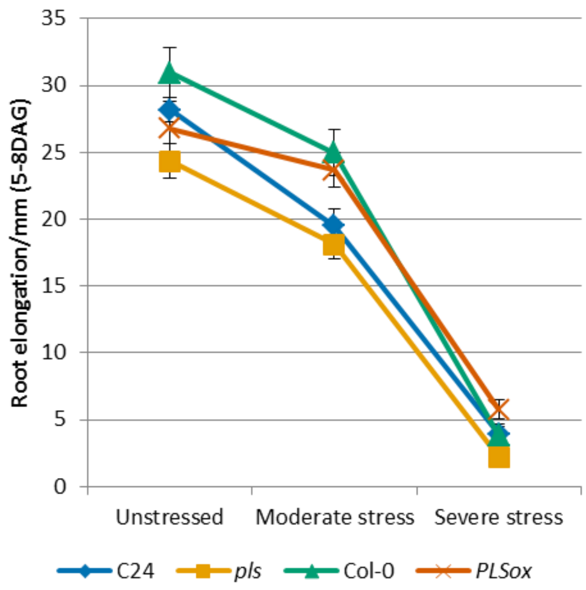

Fig. S2 The ethylene oversensitive mutant *polaris (pls)* displays similar root growth responses to wildtype (C24) under osmotic stress treatment

The ethylene oversensitive mutant *polaris (pls)* displays similar root growth responses to wildtype (C24) under osmotic stress treatment. The 35S::PLS line (PLSox) has reduced ethylene sensitivity and displays similar root growth responses to wildtype (Col-0) under osmotic stress treatment.

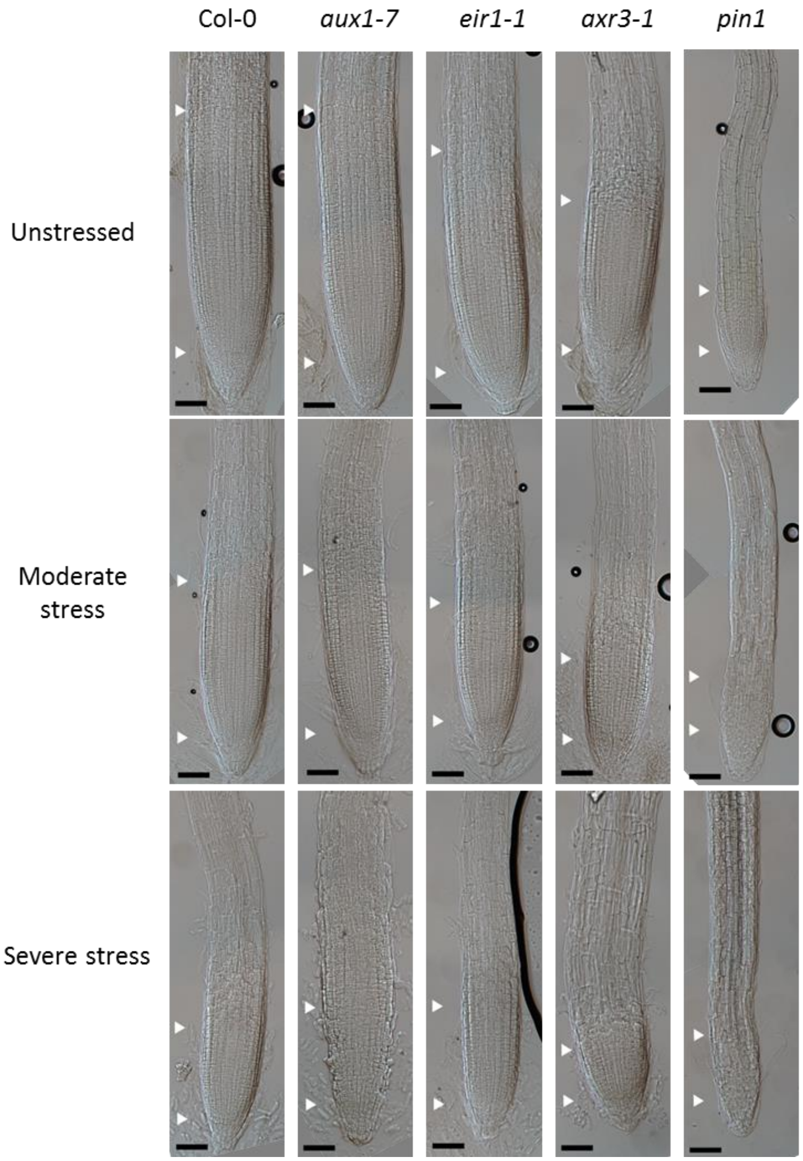

Fig. S3 Arabidopsis mutant meristems after 4 d of osmotic stress treatment

Seedlings were transferred from 1/2MS plates to 1/2MS + Poly(ethylene glycol) mol wt. 8000 at 5 d after germination (DAG). After 4 d treatment, roots were removed, mounted in Hoyer's solution and imaged under a compound microscope. Basipetal auxin transport mutants *pin2/eir1-1* and *aux1-7* display a similar reduction in meristem size under osmotic stress to wildtype (Col-0). The auxin resistant mutant *axr3-1* displays a more severe reduction in meristem size under osmotic stress. The acropetal auxin transport mutant *pin1* displays a severely reduced meristem under unstressed conditions, that does not appear to reduce in size under stress. Arrowheads indicate the position of the quiescent centre and the end of the meristematic zone. Scale bars, 50 µm.

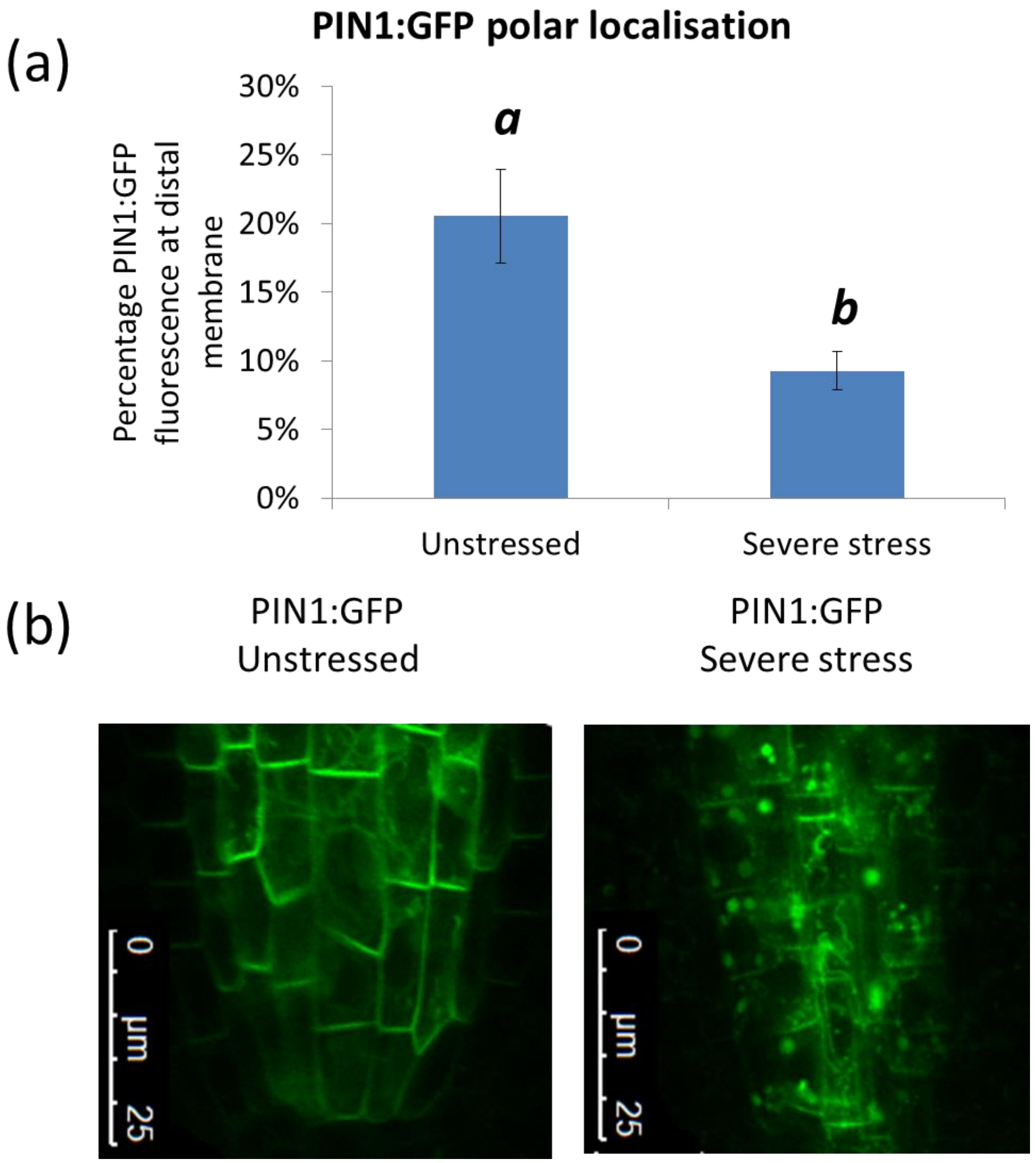

Fig. S4 PIN1 localisation changes under osmotic stress

(a) PIN1 polar localisation is reduced under osmotic stress. The data represent the percentage of fluorescence at the distal membrane, when compared to the entire cell, as measured in ImageJ. At least 10 cells in 3 different roots were measured. Lowercase letters indicate significance with a student's *T*-test, P=0.03. Error bars indicate ±SEM.
(b) PIN1 accumulates in internal bodies under osmotic stress.

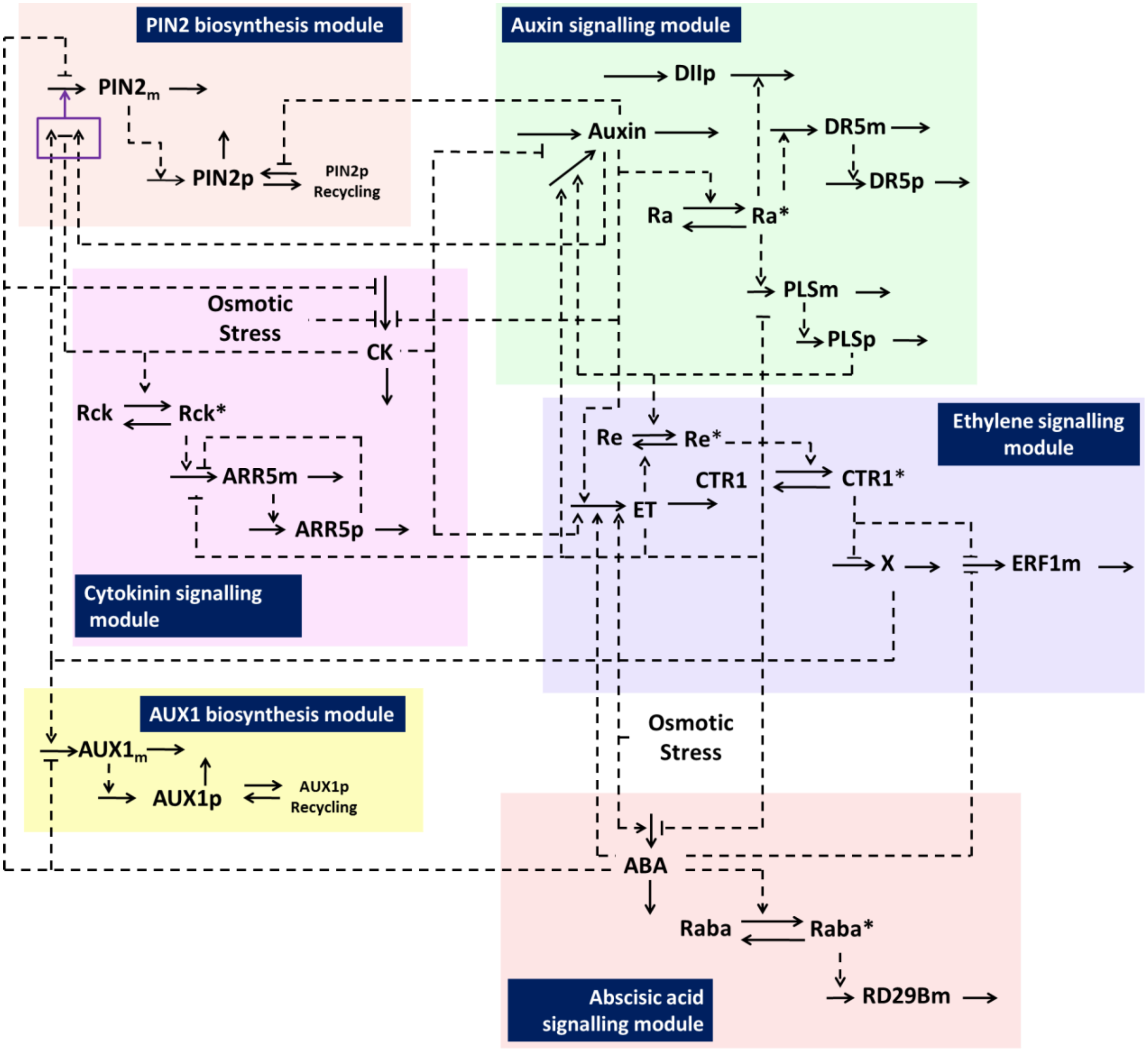

Fig. S5 A hormonal crosstalk network for the regulation of root growth under osmotic stress conditions, in an epidermis/cortex cell expressing PIN2

Symbols: Auxin, auxin; Ra, inactive auxin receptor; Ra*, active auxin receptor; DR5m, *DR5* regulated *YFP* mRNA transcript; DR5p, *DR5* regulated YFP protein; DIIp, DII-VENUS protein; PIN1m, *PIN1* mRNA transcript; PIN1p, PIN1 transporter protein; AUX1m, AUX1 mRNA transcript; AUX1p, AUX1 transporter; PLSm, *POLARIS* mRNA transcript; PLSp, POLARIS peptide; ET, ethylene; Re, inactive ethylene receptor; Re*, active ethylene receptor; CTR1, inactive CTR1 kinase; CTR1* active CTR1 kinase; X, the unknown factor that regulates auxin transport from the aerial tissues; ERF1m, *ERF1* mRNA transcript; ABA, abscisic acid; Raba, inactive abscisic acid receptor; Raba*, active abscisic acid receptor; RD29Bm, *RD29B* mRNA transcript CK, active cytokinin; Rck, inactive cytokinin receptor; Rck*, active cytokinin receptor; ARR5m, *ARR5* mRNA transcript; ARR5p, ARR5 protein; Osmotic stress, the osmotic stress imposed by the growth medium.

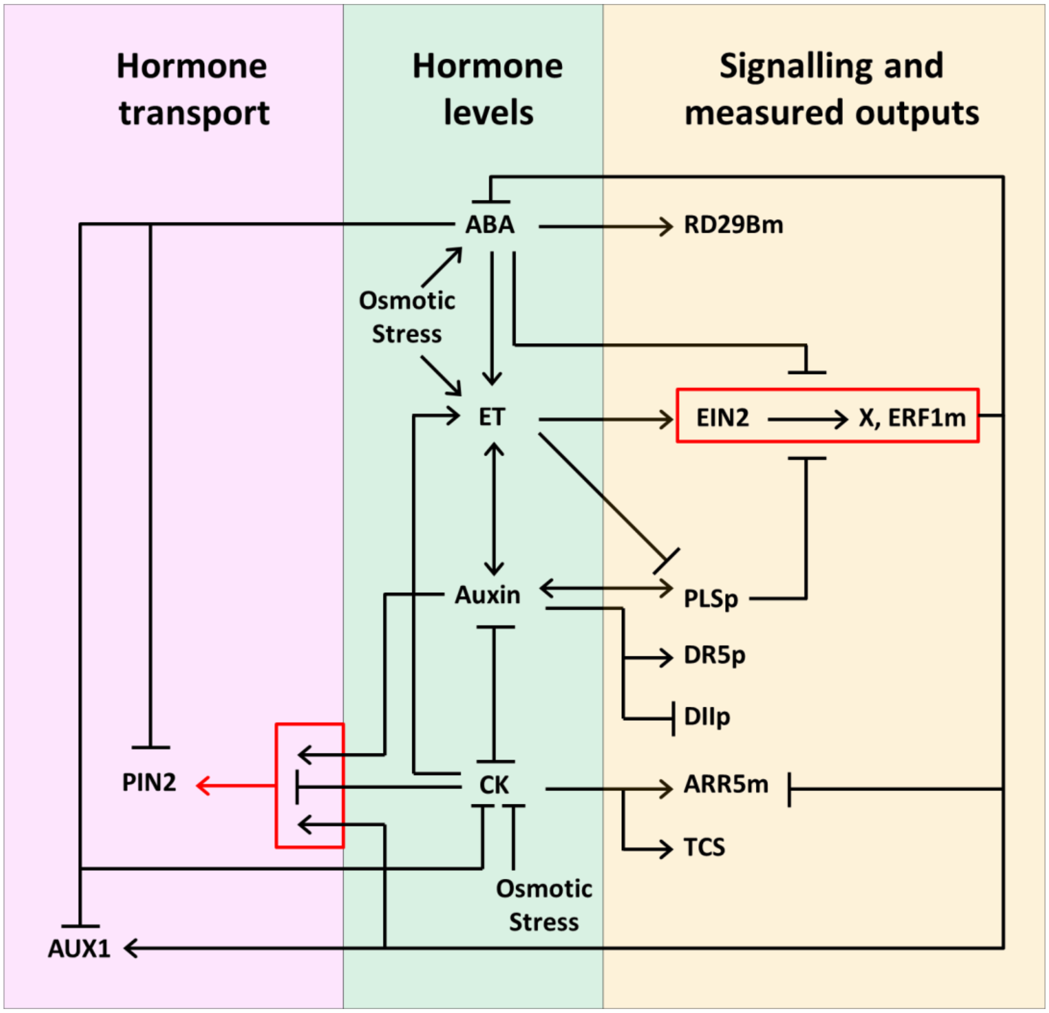

Fig. S6 A simplified schematic for the hormonal crosstalk network for the regulation of root growth in an epidermis/cortex cell expressing PIN2

Symbols: Auxin, auxin; DR5p, *DR5* regulated YFP protein; DIIp, DII-VENUS protein; PIN2, PIN2 auxin efflux transporter protein; AUX1, AUX1 auxin influx transporter protein; PLSp, POLARIS peptide; ET, ethylene; X, the unknown factor that regulates auxin transport from the aerial tissues; EIN2, EIN2 ethylene signalling protein; ERF1m, *ERF1* mRNA transcript; ABA, abscisic acid; RD29Bm, *RD29B* mRNA transcript, CK, active cytokinin; Rck, inactive cytokinin receptor; Rck*, active cytokinin receptor; Osmotic stress, the osmotic stress imposed by the growth medium. Red boxes group related activities.

Table S1 Primer sequences

Gene	Primer sequences 5'-3'	Tm (°C)	Notes
AT5G15710	CTCTTTCGCCTCTTGGTTTG<br>TCCTTCCCACGAGAAACAAT	57.3<br>55.3	Househeeping gene, selected due to stability in roots/under hormones/under abiotic stress (Czechowski *et al*., 2005)
RD29B	GGG GAA AGG ACA TGG TGA GG<br>GGT TTA CCA CCG AGC CAA GA	60.03<br>59.96	ABA dependent drought responsive gene.
DREB2B	CCC ATC AGA GCC AAG ACC AA<br>GGA CCA TTG CCT CAG AAC TC	59.67<br>58.26	ABA independent, drought responsive gene, a TF which is expressed early, primer works well for qPCR with touchdown
ARR5	TGT CCT GAT TCT TTC GGC TT<br>ACC CAT CTT TGT CAC TCT TGA	57.14<br>56.85	Consistently produces a band but with primer dimers
ERF1	GGTATTAGGGTTTGGCTCGG<br>CCGAAAGCGACTCTTGAACT	58.04<br>58.22	Ethylene responsive gene
PIN4	CGGCAACAACGGAACACATA<br>CGGTAAGCAACAAGAGCCCA	59.13<br>60.61	Auxin efflux carrier
PIN2	AATGCTGGTTGCTTTGCCTG<br>CCTTTGGGTCGTATCGCCTT	59.97<br>60.11	Auxin efflux carrier
PIN1	TCGTTGCTTCTTATGCCGTT<br>AGAAGAGTTATGGGCAACGC	58.20<br>58.26	Auxin efflux carrier
AUX1	TCTCTCGCTCACATGCTCAC<br>CGTCCAGCTCGGCATAAAGA	59.83<br>60.18	Auxin influx carrier
ACT2	GGA TCG GTG GTT CCA TTC TTGC<br>AGA GTT TGT CAC ACA CAA GTG CA	56.00<br>55.21	Used to verify samples were free of genomic DNA contamination. Produces a 256 bp cDNA band and a 342 bp genomic band

Reference

Czechowski T, Stitt M, Altmann T, Udvardi MK, Scheible WR. 2005. Genome-wide identification and testing of superior reference genes for transcript normalization in Arabidopsis. *Plant Physiology* **139**: 5–17.

Notes S1

Construction of hormonal crosstalk networks under osmotic stress conditions

We previously developed a hormonal interaction network for a single Arabidopsis cell by iteratively combining modelling with experimental analysis (Liu *et al.*, 2010). We described how such a network regulates auxin concentration in the Arabidopsis root, by controlling the relative contribution of auxin influx, biosynthesis and efflux; and by integrating auxin, ethylene and cytokinin signalling. Recently, we have developed this hormonal interaction network to include PIN1 or PIN2 activities in a single Arabidopsis cell (Liu *et al.*, 2013, 2014), and moved on to study the spatiotemporal dynamics of hormonal crosstalk in a multi-cellular root structure (Moore *et al.*, 2015). Here we show that, after now incorporating abscisic acid (ABA) into the existing hormonal crosstalk network, a novel hormonal crosstalk network for osmotic stress conditions can be constructed. Fig. 7 in the main text describes how ABA, cytokinin, ethylene, auxin, PIN1 and AUX1 interplay in a single stele cell under osmotic stress conditions. The rationale for the network construction is now described.

Abscisic acid and ethylene biosynthesis and crosstalk

Contemporary and classic studies show that ABA biosynthesis increases under osmotic stress, and is responsible for many stress responses (Wright & Hiron, 1969; Zhang & Davies, 1987; Bray, 1997; Lee *et al.*, 2006; Verslues & Bray, 2006; Jones *et al.*, 2014; Waadt *et al.*, 2014). This ABA increase is larger in shoot tissues than roots (Christmann *et al.*, 2005) but is important for root growth under stress. There are several putative osmosensors (Urao *et al.*, 1999; Reiser *et al.*, 2003; Wohlbach *et al.*, 2008; Kumar *et al.*, 2013; Yuan *et al.*, 2014) but the full signalling pathway leading to increased ABA biosynthesis is unknown. Therefore, we assume that ABA increases in response to a more negative external osmotic pressure (Fig. 7 in the main text).

There are several reports of increased ethylene biosynthesis under osmotic stress and ethylene signalling has been shown to be important in many drought stress responses (Spollen *et al.*, 2000; Skirycz *et al.*, 2011; Cheng *et al.*, 2013; Cui *et al.*, 2015). Moreover, ABA and ethylene can act either antagonistically or synergistically to affect root growth and development, but

phenotypic analysis of ethylene-ABA mutant crosses reveals little crosstalk between the signalling cascades (Cheng *et al.*, 2009). Furthermore, it has been proposed that ABA represses ethylene biosynthesis to help maintain root growth under stress (Spollen *et al.*, 2000; Sharp, 2002; Li *et al.*, 2011).

Although ABA represses the expression of ethylene response genes such as *ERF1*, as well as preventing ethylene-induced quiescent centre cell division, there is now growing evidence that ABA promotes ethylene biosynthesis to inhibit root growth (Ortega-Martinez *et al.*, 2007; Zhang *et al.*, 2010; Cheng *et al.*, 2013; Luo *et al.*, 2014). An intact ethylene signalling cascade is required for ABA inhibition of root growth and this requires ethylene-induced basipetal auxin transport components such as PIN2 and AUX1 (Beaudoin *et al.*, 2000; Ghassemian *et al.*, 2000; Luo *et al.*, 2014; Thole *et al.*, 2014).

A combination of these experimental and other observations indicates that ABA can promote ethylene biosynthesis but negatively regulates aspects of its response downstream of the main signalling cascade, such as *PIN1* and *ERF1* gene expression. In addition, ethylene insensitive/deficient mutants have been shown to hyperaccumulate ABA, so it is possible that ethylene inhibits ABA biosynthesis, providing a negative feedback (Beaudoin *et al.*, 2000; Ghassemian *et al.*, 2000; Wang *et al.*, 2007; Cheng *et al.*, 2009; Dong *et al.*, 2011).

Regulation of auxin transport by both ABA and ethylene

Whilst it has been reported that endogenously applied ABA can increase PIN2 protein levels and basipetal auxin transport in the root (Xu *et al.*, 2013), other work indicates that PIN1, PIN2 and AUX1 levels are reduced by ABA, probably through changes in *PLETHORA* gene expression (Belin *et al.*, 2009; Shkolnik-Inbar & Bar-Zvi, 2010; Yang *et al.*, 2014). Our experimental data show that AUX1 expression is repressed under osmotic stress (Fig. 5). Thus, in the hormonal crosstalk network (Fig. 7 in the main text), we consider *AUX1* expression to be repressed by ABA.

In addition, the overriding effects of ABA on the regulation of PIN1 by ethylene imply that ABA acts on components downstream of ethylene signalling. We therefore assume that ABA

negatively regulates PIN1 downstream of the ethylene signalling cascade (Fig. 7 in the main text). This overriding mechanism would allow PIN1 levels to decrease under osmotic stress, even as ethylene levels increase.

However, the regulation of auxin efflux carriers by ABA is tissue-specific. PIN1, which is expressed in the stele, and PIN2, which is expressed in the epidermis/cortex cells, differentially respond to osmotic stress (Fig. 5 in the main text). Moreover, PIN2 protein accumulation can increase under osmotic stress due to the ethylene response (Fig. 5 in the main text), implying that ABA does not override the regulation of PIN2 by ethylene. Therefore, the crosstalk between PIN2, ABA and ethylene is different from the crosstalk between PIN1, ABA and ethylene, as described in Fig. S4. In addition, decreased PIN1 protein levels and increased PIN2 protein levels (Fig. 5 in the main text) can explain reduced meristem auxin levels under osmotic stress.

Cytokinin, abscisic acid and osmotic stress

Drought and ABA negatively affect *trans*-zeatin-type cytokinin levels by modulating expression of cytokinin biosynthesis/metabolism enzymes (Dobra *et al.*, 2010; Nishiyama *et al.*, 2011). As it is unclear whether osmotic stress affects cytokinin levels directly or through ABA signalling, our networks (Figs 7 (in the main text), S4) have included both effects that can limit cytokinin biosynthesis.

Cytokinin deficient/insensitive mutants display reduced ABA levels but increased ABA sensitivity, and drought induction of ABA biosynthesis has been shown to be similar to wildtype (Nishiyama *et al.*, 2011). Lower basal levels of ABA could either be due to an increase in auxin and ethylene signalling in these mutants, suppressing ABA biosynthesis or cytokinin could be directly regulating ABA biosynthesis. Thus, no direct regulation of ABA biosynthesis by cytokinin is considered in the hormonal crosstalk networks (Figs 7 (in the main text), S4).

Gene expression

Expression of the relevant genes we have studied has been included in the hormonal crosstalk networks (Figs 7 (in the main text), S4). These genes are either hormone signalling reporter constructs (DR5 and DII VENUS for auxin) or the components important for hormonal crosstalk (PIN1, PIN2, AUX1 and PLS). Inclusion of gene expression enables us to establish the relationship between hormones, gene expression and osmotic stress.

Hormonal crosstalk networks for a single stele cell and a single epidermis/cortex cell

By integrating the biological knowledge described above with the hormonal crosstalk network we previously developed, a hormonal crosstalk network can be constructed for a single stele cell (Fig. 7 in the main text) and a single epidermis/cortex cell (Fig. S4), respectively. The hormonal crosstalk networks describe how ABA, cytokinin, ethylene, auxin, PIN1 or PIN2, and AUX1 interplay in a single cell under osmotic stress conditions. The key difference between Fig. 7 in the main text and Fig. S4 is ABA could override ethylene induction of *PIN1* gene expression, whilst still allowing *PIN2* expression to increase.

References

Beaudoin N, Serizet C, Gosti F, Giraudat J. 2000. Interactions between abscisic acid and ethylene signaling cascades. *The Plant Cell* **12**: 1103–1115.

Belin C, Megies C, Hauserova E, Lopez-Molina L. 2009. Abscisic acid represses growth of the arabidopsis embryonic axis after germination by enhancing auxin signaling. *The Plant Cell* **21**: 2253–2268.

Bray EA. 1997. Plant responses to water deficit. *Trends in Plant Science* **2**: 48–54.

Cheng M-C, Liao P-M, Kuo W-W, Lin T-P. 2013. The Arabidopsis ETHYLENE RESPONSE FACTOR1 regulates abiotic stress-responsive gene expression by binding to different cis-acting elements in response to different stress signals. *Plant Physiology* **162**: 1566–1582.

Cheng W-H, Chiang M-H, Hwang S-G, Lin P-C. 2009. Antagonism between abscisic acid and ethylene in Arabidopsis acts in parallel with the reciprocal regulation of their metabolism and signaling pathways. *Plant Molecular Biology* **71**: 61–80.

Christmann A, Hoffmann T, Teplova I, Grill E, Muller A. 2005. Generation of active pools of abscisic acid revealed by *in vivo* imaging of water-stressed Arabidopsis. *Plant Physiology* **137**: 209–219.

Cui M, Lin Y, Zu Y, Efferth T, Li D, Tang Z. 2015. Ethylene increases accumulation of compatible solutes and decreases oxidative stress to improve plant tolerance to water stress in Arabidopsis. *Journal of Plant Biology* **58**: 193–201.

Dobra J, Motyka V, Dobrev P, Malbeck J, Prasil IT, Haisel D, Gaudinova A, Havlova M, Gubis J, Vankova R. 2010. Comparison of hormonal responses to heat, drought and combined stress in tobacco plants with elevated proline content. *Journal of Plant Physiology* **167**: 1360–1370.

Dong H, Zhen Z, Peng J, Chang L, Gong Q, Wang NN. 2011. Loss of ACS7 confers abiotic stress tolerance by modulating ABA sensitivity and accumulation in Arabidopsis. *Journal of Experimental Botany* **62**: 4875–4887.

Ghassemian M, Nambara E, Cutler S, Kawaide H, Kamiya Y, McCourt P. 2000. Regulation of abscisic acid signaling by the ethylene response pathway in arabidopsis. *The Plant Cell* **12**: 1117–1126.

Jones AM, Danielson JÅ, ManojKumar SN, Lanquar V, Grossmann G, Frommer WB. 2014. Abscisic acid dynamics in roots detected with genetically encoded FRET sensors. *Elife* **3**: e01741.

Kumar MN, Jane W-N, Verslues PE. 2013. Role of the putative osmosensor Arabidopsis HISTIDINE KINASE1 in dehydration avoidance and low-water-potential response. *Plant Physiology* **161**: 942–953.

Lee KH, Piao HL, Kim H-Y, Choi SM, Jiang F, Hartung W, Hwang I, Kwak JM, Lee I-J, Hwang I. 2006. Activation of glucosidase via stress-induced polymerization rapidly increases active pools of abscisic acid. *Cell* **126**: 1109–1120.

Li Z, Zhang L, Yu Y, Quan R, Zhang Z, Zhang H, Huang R. 2011. The ethylene response factor AtERF11 that is transcriptionally modulated by the bZIP transcription factor HY5 is a crucial repressor for ethylene biosynthesis in Arabidopsis. *The Plant Journal* **68**: 88–99.

Liu J, Mehdi S, Topping J, Friml J, Lindsey K. 2013. Interaction of PLS and PIN and hormonal crosstalk in Arabidopsis root development. *Frontiers in Plant Science* **4**: 75.

Liu J, Mehdi S, Topping J, Tarkowski P, Lindsey K. 2010. Modelling and experimental analysis of hormonal crosstalk in Arabidopsis. *Molecular Systems Biology* **6**: 373.

Liu J, Rowe J, Lindsey K. 2014. Hormonal crosstalk for root development: a combined experimental and modeling perspective. *Frontiers in Plant Science* **5**: 116.

Luo X, Chen Z, Gao J, Gong Z. 2014. Abscisic acid inhibits root growth in Arabidopsis through ethylene biosynthesis. *The Plant Journal* **79**: 44–55.

Moore S, Zhang X, Mudge A, Rowe JH, Topping JF, Liu J, Lindsey K. 2015. Spatiotemporal modelling of hormonal crosstalk explains the level and patterning of hormones and gene expression in *Arabidopsis thaliana* wild-type and mutant roots. *New Phytologist* **207**: 1110–1122.

Nishiyama R, Watanabe Y, Fujita Y, Dung Tien L, Kojima M, Werner T, Vankova R, Yamaguchi-Shinozaki K, Shinozaki K, Kakimoto T *et al*. 2011. Analysis of cytokinin mutants and regulation of cytokinin metabolic genes reveals important regulatory roles of cytokinins in drought, salt and abscisic acid responses, and abscisic acid biosynthesis. *The Plant Cell* **23**: 2169–2183.

Ortega-Martinez O, Pernas M, Carol RJ, Dolan L. 2007. Ethylene modulates stem cell division in the *Arabidopsis thaliana* root. *Science* **317**: 507–510.

Reiser V, Raitt DC, Saito H. 2003. Yeast osmosensor Sln1 and plant cytokinin receptor Cre1 respond to changes in turgor pressure. *Journal of Cell Biology* **161**: 1035–1040.

Sharp RE. 2002. Interaction with ethylene: changing views on the role of abscisic acid in root and shoot growth responses to water stress. *Plant, Cell & Environment* **25**: 211–222.

Shkolnik-Inbar D, Bar-Zvi D. 2010. ABI4 mediates abscisic acid and cytokinin inhibition of lateral root formation by reducing polar auxin transport in Arabidopsis. *The Plant Cell* **22**: 3560–3573.

Skirycz A, Claeys H, De Bodt S, Oikawa A, Shinoda S, Andriankaja M, Maleux K, Eloy NB, Coppens F, Yoo S-D *et al*. 2011. Pause-and-stop: the effects of osmotic stress on cell proliferation during early leaf development in Arabidopsis and a role for ethylene signaling in cell cycle arrest. *The Plant Cell* **23**: 1876–1888.

Spollen WG, LeNoble ME, Samuels TD, Bernstein N, Sharp RE. 2000. Abscisic acid accumulation maintains maize primary root elongation at low water potentials by restricting ethylene production. *Plant Physiology* **122**: 967–976.

Thole JM, Beisner ER, Liu J, Venkova SV, Strader LC. 2014. Abscisic acid regulates root elongation through the activities of auxin and ethylene in *Arabidopsis thaliana*. *G3-Genes Genomes Genetics* **4**: 1259–1274.

Urao T, Yakubov B, Satoh R, Yamaguchi-Shinozaki K, Seki M, Hirayama T, Shinozaki K. 1999. A transmembrane hybrid-type histidine kinase in arabidopsis functions as an osmosensor. *The Plant Cell* **11**: 1743–1754.

Verslues PE, Bray EA. 2006. Role of abscisic acid (ABA) and *Arabidopsis thaliana* ABA-insensitive loci in low water potential-induced ABA and proline accumulation. *Journal of Experimental Botany* **57**: 201–212.

Waadt R, Hitomi K, Nishimura N, Hitomi C, Adams SR, Getzoff ED, Schroeder JI. 2014. FRET-based reporters for the direct visualization of abscisic acid concentration changes and distribution in Arabidopsis. *Elife* **3**: e01739.

Wang Y, Liu C, Li K, Sun F, Hu H, Li X, Zhao Y, Han C, Zhang W, Duan Y *et al*. 2007. Arabidopsis EIN2 modulates stress response through abscisic acid response pathway. *Plant Molecular Biology* **64**: 633–644.

Wohlbach DJ, Quirino BF, Sussman MR. 2008. Analysis of the Arabidopsis histidine kinase ATHK1 reveals a connection between vegetative osmotic stress sensing and seed maturation. *The Plant Cell* **20**: 1101–1117.

Wright STC, Hiron RWP. 1969. (+)-Abscisic acid, growth inhibitor induced in detached wheat leaves by a period of wilting. *Nature* **224**: 719.

Xu W, Jia L, Shi W, Liang J, Zhou F, Li Q, Zhang J. 2013. Abscisic acid accumulation modulates auxin transport in the root tip to enhance proton secretion for maintaining root growth under moderate water stress. *New Phytologist* **197**: 139–150.

Yang L, Zhang J, He J, Qin Y, Hua D, Duan Y, Chen Z, Gong Z. 2014. ABA-mediated ros in mitochondria regulate root meristem activity by controlling *PLETHORA* expression in Arabidopsis. *Plos Genetics* **10,** e1004791.

Yuan F, Yang H, Xue Y, Kong D, Ye R, Li C, Zhang J, Theprungsirikul L, Shrift T, Krichilsky B *et al*. 2014. OSCA1 mediates osmotic-stress-evoked Ca^{2+} increases vital for osmosensing in Arabidopsis. *Nature* **514**: 367–371.

Zhang H, Han W, De Smet I, Talboys P, Loya R, Hassan A, Rong H, Juergens G, Knox JP, Wang M-H. 2010. ABA promotes quiescence of the quiescent centre and suppresses

stem cell differentiation in the Arabidopsis primary root meristem. *The Plant Journal* **64**: 764–774.

Zhang J, Davies WJ. 1987. Increased synthesis of ABA in partially dehydrated root-tips and ABA transport from roots to leaves. *Journal of Experimental Botany* **38**: 2015–2023.
